# Chronic pain–induced neuronal plasticity in the bed nucleus of the stria terminalis causes maladaptive anxiety

**DOI:** 10.1126/sciadv.abj5586

**Published:** 2022-04-27

**Authors:** Naoki Yamauchi, Keiichiro Sato, Kenta Sato, Shunsaku Murakawa, Yumi Hamasaki, Hiroshi Nomura, Taiju Amano, Masabumi Minami

**Affiliations:** Department of Pharmacology, Graduate School of Pharmaceutical Sciences, Hokkaido University, Sapporo 060-0812, Japan.

## Abstract

The comorbidity of chronic pain and mental dysfunctions such as depression and anxiety disorders has long been recognized, but the underlying mechanisms remain poorly understood. Here, using a mouse model of neuropathic pain, we demonstrated neuronal plasticity in the bed nucleus of the stria terminalis (BNST), which plays a critical role in chronic pain–induced maladaptive anxiety. Electrophysiology demonstrated that chronic pain increased inhibitory inputs to lateral hypothalamus (LH)–projecting BNST neurons. Chemogenetic manipulation revealed that sustained suppression of LH-projecting BNST neurons played a crucial role in chronic pain–induced anxiety. Furthermore, using a molecular genetic approach, we demonstrated that chronic pain elevated the excitability of a specific subpopulation of BNST neurons, which express cocaine- and amphetamine-regulated transcript (CART). The elevated excitability of CART-positive neurons caused the increased inhibitory inputs to LH-projecting BNST neurons, thereby inducing anxiety-like behavior. These findings shed light on how chronic pain induces psychiatric disorders, characterized by maladaptive anxiety.

## Teaser

Neuronal plasticity in the limbic circuit causes maladaptive anxiety in a mouse model of neuropathic pain.

## INTRODUCTION

Pain is essential for our survival because it functions as a biological alarm for impending or actual tissue damage. However, chronic pain is a persistent, inescapable stress, which leads to maladaptive emotional states (*1*). It is often comorbid with psychiatric disorders, such as depression and anxiety disorders (*2*–*4*). Preclinical studies have reported depression- and anxiety-like behaviors in animal models of chronic pain (*5*–*8*). These findings suggest that chronic pain causes plastic changes in neural circuits and gives rise to negative emotions, such as depression and anxiety. However, the brain areas of neuronal plasticity that lead to psychiatric disorders remain to be elucidated.

The bed nucleus of the stria terminalis (BNST) is a forebrain structure implicated in negative emotional states, such as anxiety and fear (*9*). This brain region receives direct and indirect nociceptive information from the spinal dorsal horn (*10*, *11*) and reportedly plays a critical role in the affective consequences of acute noxious stimuli (*12*–*14*). Our recent study demonstrated that chronic pain increased inhibitory inputs to BNST output neurons projecting to the ventral tegmental area (VTA) and thereby suppressed VTA dopaminergic neuron activity (*15*). However, it remains unclear whether increased inhibitory inputs to BNST output neurons are involved in chronic pain–induced anxiety-like behavior, and what is the neuronal mechanism for the increased inhibitory inputs during chronic pain. To address these issues, we performed behavioral and electrophysiological analyses using chemogenetic and optogenetic techniques to manipulate the neuronal activity of a specific population of BNST neurons. Since selective activation of the anterodorsal BNST (adBNST) output neurons projecting to the lateral hypothalamus (LH) has been reported to produce an anxiolytic effect (*16*), we examined whether the persistent increase in inhibitory inputs to LH-projecting adBNST neurons was involved in anxiety-like behavior observed in chronic pain model mice. Reportedly, most BNST neurons are GABAergic (*17*). Therefore, we also investigated whether some populations of BNST neurons had inhibitory synapses onto LH-projecting adBNST neurons, and whether chronic pain elevated the excitability of these inhibitory neurons. The results suggest that chronic pain elevated the excitability of a genetically identified subpopulation of BNST GABAergic neurons, thereby increasing the inhibitory inputs to LH-projecting adBNST neurons, which induced anxiety-like behavior. The current study sheds light on the neuronal mechanisms underlying psychiatric disorders associated with chronic pain.

## RESULTS

### Chronic pain enhances anxiety-like behavior

**Fig. 1.**
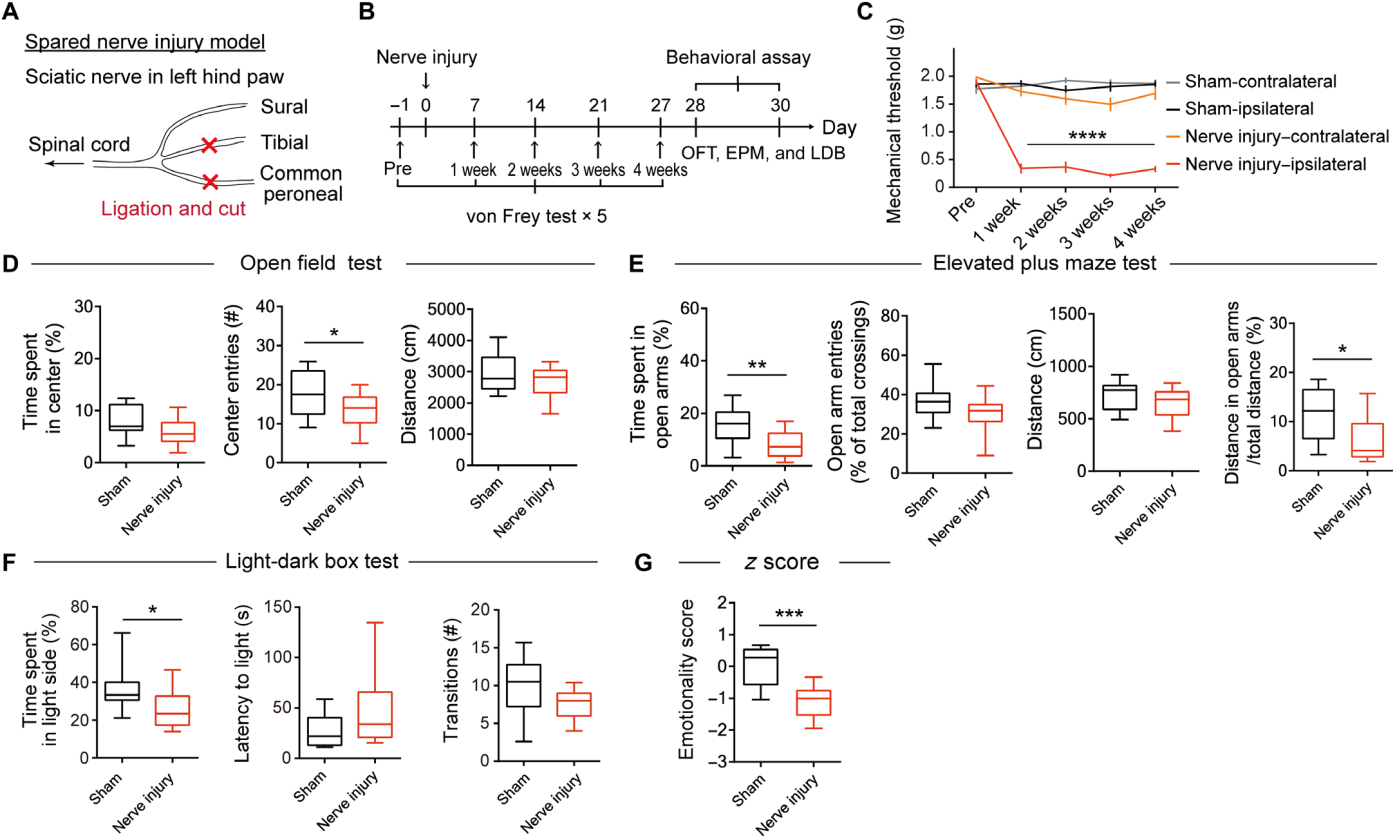
Chronic pain induces anxiety-like behavior. (**A**) Spared nerve injury model. Nerve injuries were induced by ligating and cutting of the tibial and common peroneal nerves, leaving the sural nerve intact. (**B**) Schedule of experiments. (**C**) Mechanical threshold of sham-operated control and nerve injury mice (sham: *n* = 23; nerve injury: *n* = 22). Data are expressed as means ± SEM. Statistical significance was evaluated using two-way repeated-measures analysis of variance (ANOVA) followed by Bonferroni’s post hoc test. ****P < 0.0001. (**D** to **F**) Behavioral analyses using OFT (D; sham: *n* = 20; nerve injury: *n* = 19), EPM (E; sham: *n* = 19; nerve injury: *n* = 18), and LDB (F; sham: *n* = 12; nerve injury: *n* = 12). (**G**) Emotionality *z* scores were calculated using the data from animals that were subjected to all three tests (sham: *n* = 9; nerve injury: *n* = 9). Box-whisker plots show the median, interquartile range, and 10th to 90th percentiles. Statistical significance was evaluated using two-tailed unpaired Student’s *t* test. *P < 0.05; **P < 0.01; ***P < 0.001. Details of statistical data are provided in table S1.

We first examined the effects of chronic pain on anxiety-like behavior using a mouse model of neuropathic pain, prepared by ligating and then cutting the tibial and common peroneal nerves on the left side (Fig. 1A). The von Frey test was performed 1 day before and weekly after the surgery to confirm the induction of chronic pain (Fig. 1B). Mice with nerve injury had a decreased threshold to mechanical stimuli at the injured (ipsilateral) hind paw over 4 weeks (Fig. 1C), indicating the presence of mechanical allodynia. Four weeks after the surgery, anxiety-like behavior was examined using the open field (OFT), elevated plus maze (EPM), and light-dark box (LDB) tests. The number of entries to the center area in the OFT (Fig. 1D), time spent in open arms in the EPM (Fig. 1E), and time spent in the light side in the LDB (Fig. 1F) were lower in the nerve injury mice, indicating enhanced anxiety-like behavior. In the EPM test, the ratio of the distance traveled in the open arms to the total distance traveled was significantly lower in the nerve injury group than in the sham group (Fig. 1E), suggesting that the decrease in exploration in open arms was not due to a decrease in locomotor activity. We calculated an emotionality *z* score to assess the consistency of behaviors across these tests. The emotionality *z* score of the nerve injury group was significantly lower than that of the sham group (Fig. 1G and fig. S1, B to D), indicating an enhancement of anxiety-like behavior by chronic pain. In addition, in the novelty-suppressed feeding (NSF) test, the latency to bite a food pellet was increased in the nerve injury mice compared with the sham-operated and naïve mice, also indicating an enhancement of anxiety-like behavior (fig. S2).

### Chronic pain increases inhibitory inputs to BNST neurons projecting to the LH

**Fig. 2.**
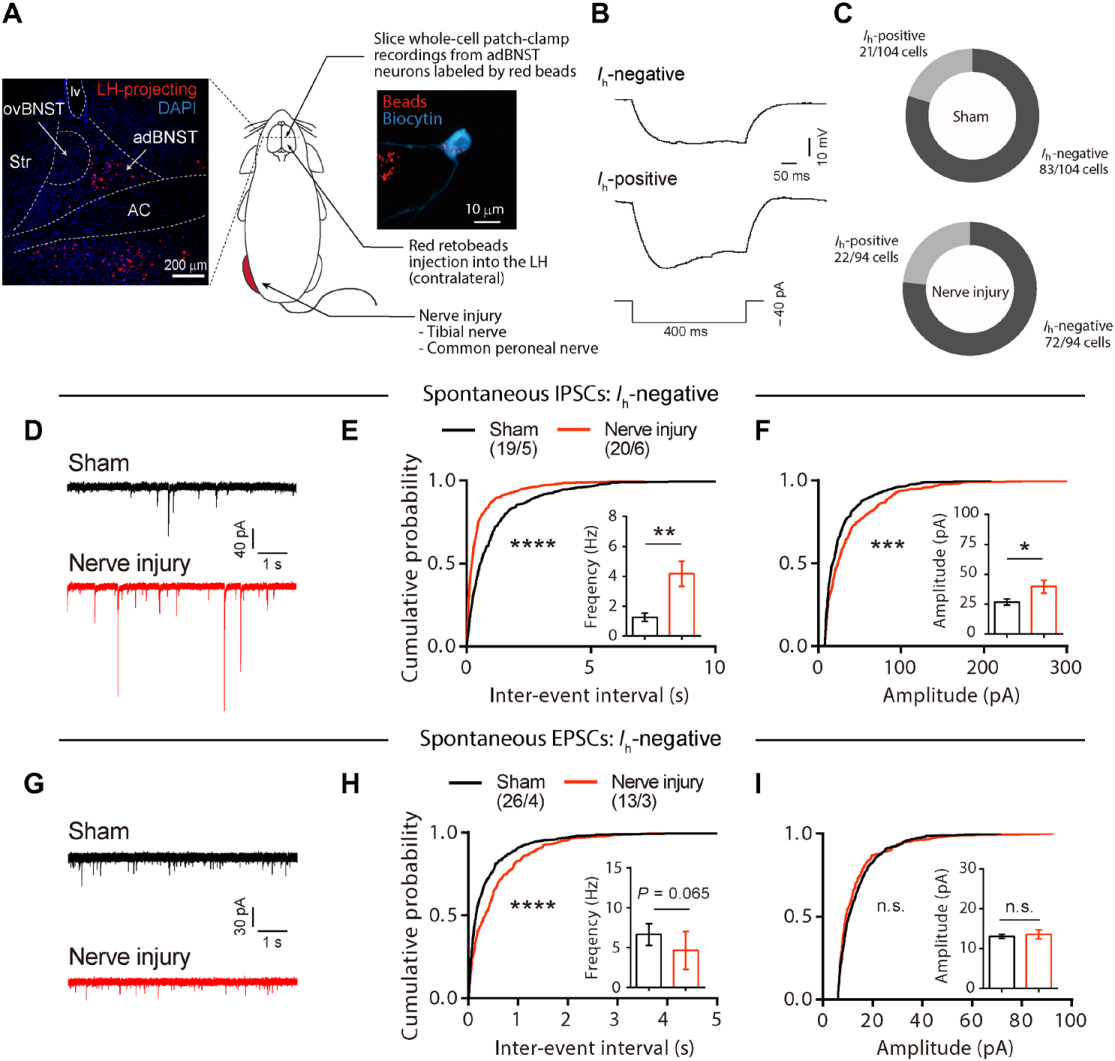
Chronic pain increases inhibitory synaptic inputs to LH-projecting adBNST neurons. (**A**) Scheme of experiments. Inserted confocal images show retrograde-labeled LH-projecting neurons (red) in the BNST (left) and a biocytin-labeled (cyan) recorded adBNST neuron (right) filled with retrobeads (red). Scale bars, 200 and 10 μm. (**B**) Representative traces of membrane potential in response to a hyperpolarizing current injection (−40 pA, 400-ms duration) recorded from *I*_h_-negative (top) and *I*_h_-positive (bottom) neurons. (**C**) Proportion of *I*_h_-negative and *I*_h_-positive neurons in LH-projecting adBNST neurons of sham-operated (top) and nerve injury (bottom) mice (*P* = 0.58, sham versus nerve injury, chi-square test). (**D**) Representative traces of sIPSCs from *I*_h_-negative LH-projecting adBNST neurons of sham-operated (top) and nerve injury (bottom) mice. (**E** and **F**) Cumulative probability plots of the inter-event interval and means ± SEM of the sIPSC frequency (E) and amplitude (F). (**G**) Representative traces of spontaneous excitatory postsynaptic currents (sEPSCs) from *I*_h_-negative LH-projecting adBNST neurons of sham-operated (top) and nerve injury (bottom) mice. (**H** and **I**) Cumulative probability plots of the inter-event interval and means ± SEM of the sEPSC frequency (H) and amplitude (I). Kolmogorov-Smirnov test was used to analyze cumulative probability plots. Two-tailed unpaired Student’s *t* test or two-tailed Mann-Whitney test was used to analyze the frequency/amplitude of sIPSC/sEPSC. *P < 0.05; **P < 0.01; ***P < 0.001; ****P < 0.0001; n.s., not significant. Details of statistical data are provided in table S1.

To investigate the synaptic inputs to LH-projecting BNST neurons, retrograde-transported fluorescent latex beads (retrobeads) were injected into the LH contralateral to the injured hind paw (Fig. 2A). LH-projecting BNST neurons were mainly observed in adBNST, as previously reported (*16*, *18*). Whole-cell patch-clamp recordings were performed on the contralateral adBNST neurons labeled with retrobeads. Sham-operated mice injected with retrobeads into the LH were used as controls. We previously reported that approximately 80% of LH-projecting dorsolateral BNST neurons in rats were hyperpolarization-activated cation current (*I*_h_) negative (*19*). Similarly, the majority of LH-projecting adBNST neurons were *I*_h_ negative in mice: 79.8 and 76.6% of the LH-projecting adBNST neurons were *I*_h_ negative in sham-operated and nerve injury mice, respectively (Fig. 2, B and C). The chi-square test showed no significant differences between the groups (χ^2^ = 0.30 and *P* = 0.55), indicating that nerve injury did not alter the proportion of *I*_h_-positive to *I*_h_-negative neurons.

In *I*_h_-negative LH-projecting adBNST neurons, the frequency and amplitude of spontaneous inhibitory postsynaptic currents (sIPSCs) were significantly increased in the nerve injury mice compared with the sham-operated mice (Fig. 2, D to F). The frequency and amplitude of sIPSCs were also increased in the *I*_h_-negative LH-projecting adBNST neurons in the ipsilateral hemisphere (fig. S3, A to D), indicating that unilateral nerve injury causes bilateral changes in inhibitory synaptic inputs to LH-projecting adBNST neurons. In *I*_h_-positive LH-projecting adBNST neurons, no differences were observed in the frequency and amplitude of sIPSCs between the sham-operated and nerve injury mice (fig. S4, A to C). Moreover, in sham-operated mice, the frequency of sIPSCs was significantly higher in *I*_h_-positive adBNST neurons than in *I*_h_-negative adBNST neurons (fig. S4D). These findings suggest a distinct inhibitory regulation between *I*_h_-negative and *I*_h_-positive adBNST neurons. The frequency and amplitude of miniature IPSCs (mIPSCs) in *I*_h_-negative LH-projecting adBNST neurons were not significantly different between nerve injury and sham-operated mice (fig. S5, A to C), indicating that the increased inhibitory synaptic inputs observed in the *I*_h_-negative LH-projecting adBNST neurons is dependent on the intra-BNST network activity. On the other hand, the frequency of spontaneous excitatory postsynaptic currents (sEPSCs) was slightly decreased in the nerve injury mice, without any changes in the amplitude (Fig. 2, G to I). These data demonstrated that chronic pain increased the inhibitory synaptic inputs to *I*_h_-negative adBNST neurons projecting to the LH.

### Inhibition of LH-projecting adBNST neurons enhances anxiety-like behavior

**Fig. 3.**
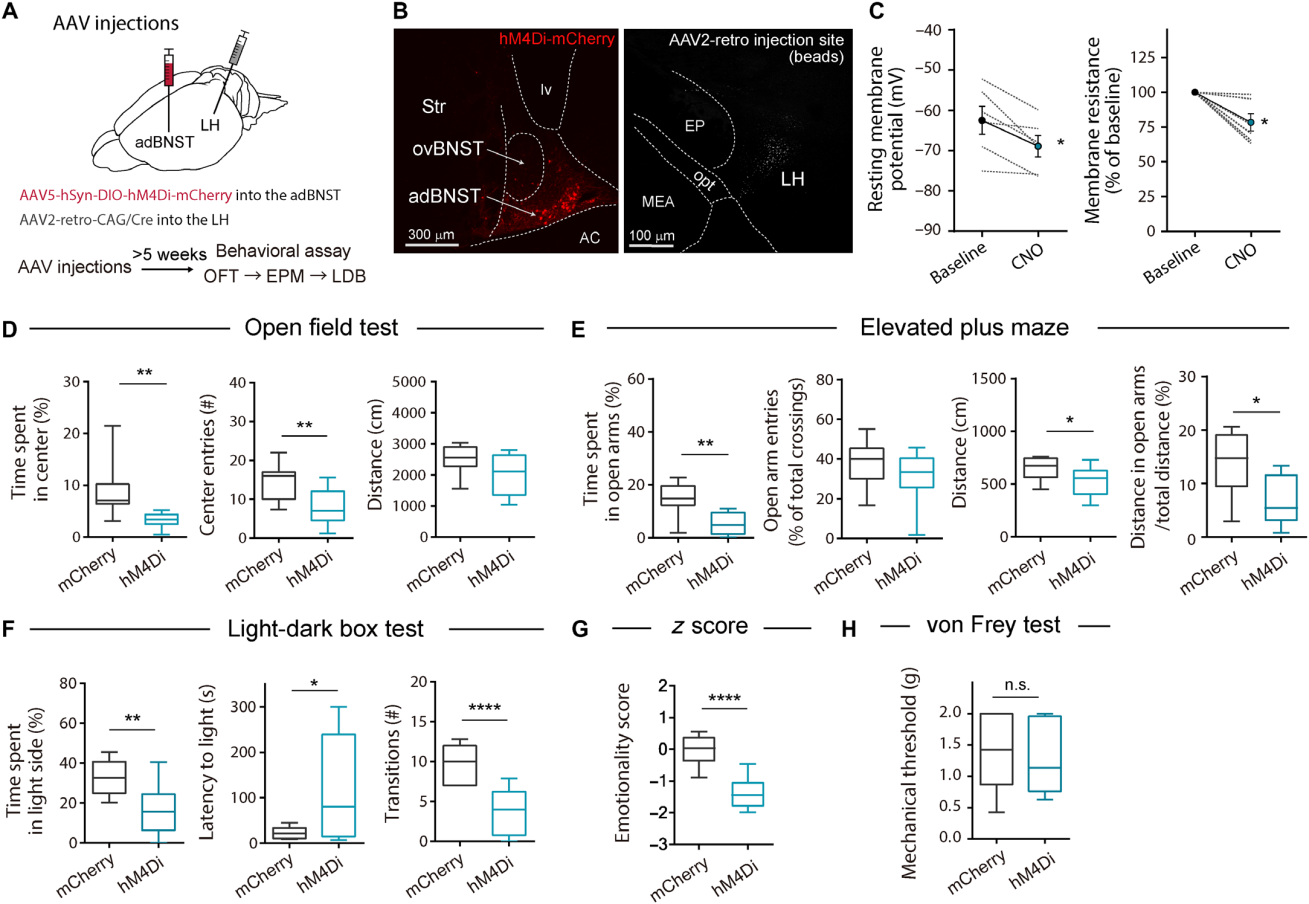
Inhibition of LH-projecting adBNST neurons induces anxiety-like behavior. (**A**) Scheme of experiments. (**B**) Representative images of hM4Di-mCherry expression in the adBNST (left) and AAV2 injection site visualized by coinjection of fluorescent beads in the LH (right). Scale bars, 300 μm (left) and 100 μm (right). AC, anterior commissure; EP, entopeduncular nucleus; lv, lateral ventricle, MEA, medial amygdala; opt, optic tract; Str, striatum. (**C**) Voltage-clamp recordings of resting membrane potential and membrane resistance in hM4Di-expressing adBNST neurons. Data are expressed as means ± SEM (*n* = 6 cells from 4 mice). Statistical significance was evaluated using two-tailed paired Student’s *t* test. *P < 0.05. (**D** to **G**) Behavioral analyses using OFT (D), EPM (E), and LDB (F), and emotionality *z* score across these tests (G). (**H**) Mechanical thresholds in the von Frey test. Box-whisker plots show the median, interquartile range, and 10th to 90th percentiles. Statistical significance was evaluated using two-tailed unpaired Student’s *t* test or two-tailed Mann-Whitney test (mCherry: *n* = 11; hM4Di: *n* = 10). *P < 0.05; **P < 0.01; ****P < 0.0001. Details of statistical data are provided in table S1.

To test whether inhibition of LH-projecting adBNST neurons enhanced anxiety-like behavior in naïve mice, we bilaterally infused retrograde-transported adeno-associated virus (AAV) 2 encoding Cre recombinase into the LH, and AAV5 carrying a Cre-dependent hM4Di-mCherry construct into the adBNST (Fig. 3, A and B). We also infused the same AAV2 into the LH and AAV5 carrying a Cre-dependent mCherry alone construct into the adBNST as controls. Histological analysis confirmed hM4Di-mCherry expression in Cre-positive neurons in the adBNST (fig. S6, A to C). Electrophysiological recordings from the hM4Di-mCherry–positive neurons showed that a bath application of clozapine-N-oxide (CNO; 10 μM) successfully hyperpolarized hM4Di-expressing adBNST neurons (Fig. 3C).

To assess the effects of inhibiting LH-projecting adBNST neurons on anxiety-like behavior, the mice were subjected to a battery of behavioral tests at least 5 weeks after AAV injections. The mice received an intraperitoneal injection of CNO (1 mg/kg) 40 min before the start of the behavioral tests. Time spent in the center area in the OFT (Fig. 3D), time spent in open arms in the EPM (Fig. 3E), and time spent in the light side in the LDB (Fig. 3F) were shorter in the mice expressing hM4Di-mCherry compared with the control group, indicating the enhancement of anxiety-like behavior. In the EPM test, the ratio of the distance traveled in the open arms to the total distance traveled was significantly lower in the nerve injury group than in the sham group (Fig. 3E), suggesting that the decrease in exploration in open arms was not due to a decrease in locomotor activity. Emotionality *z* score also showed significant enhancement of anxiety-like behavior in the hM4Di-mCherry group (Fig. 3G and fig. S7, B to D). No significant differences were observed in the threshold to mechanical stimuli in the von Frey test (Fig. 3H).

### Activation of LH-projecting adBNST neurons ameliorates chronic pain–induced anxiety-like behavior

**Fig. 4.**
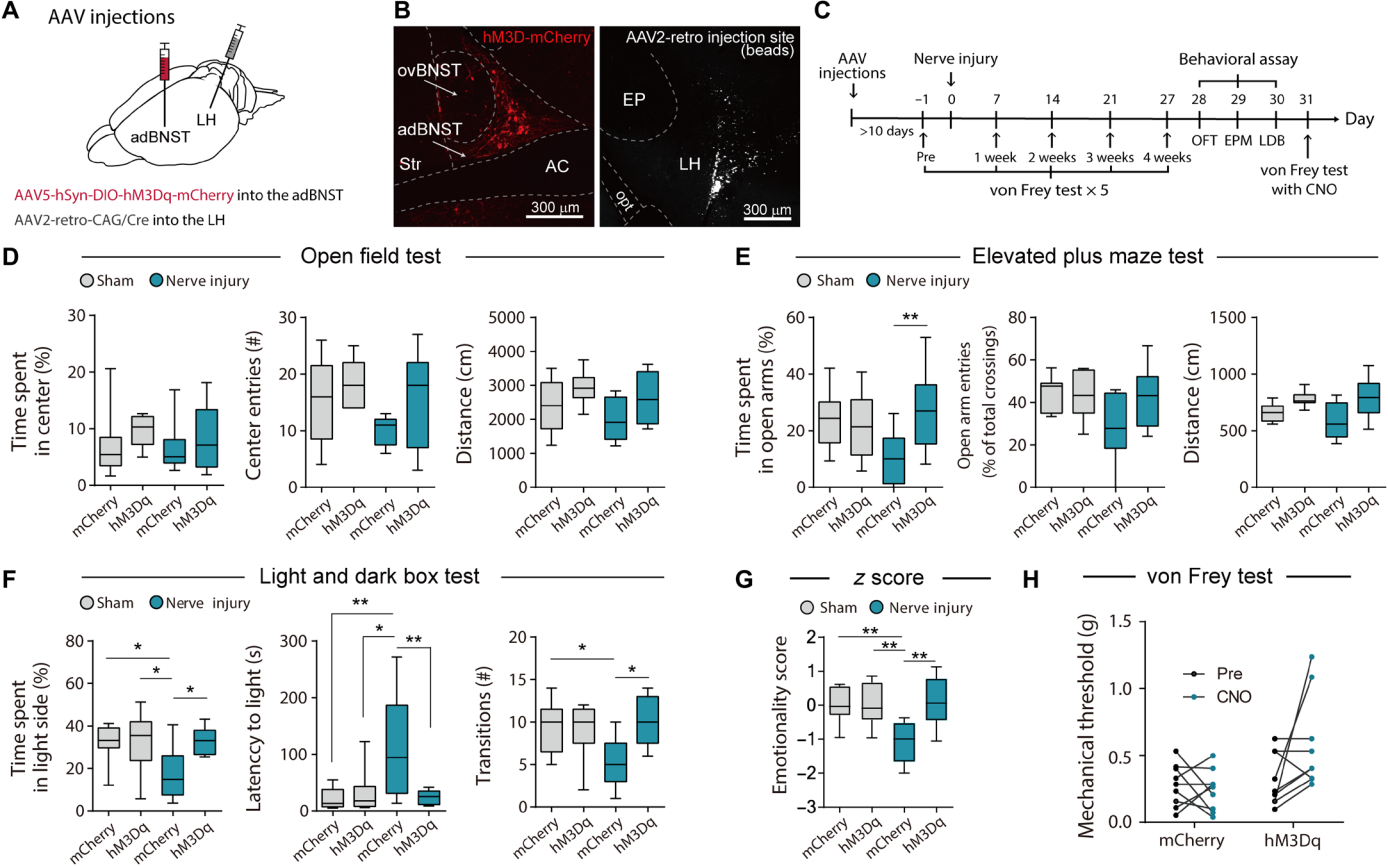
Activation of LH-projecting adBNST neurons ameliorates chronic pain–induced anxiety-like behavior. (**A**) Scheme of experiments. (**B**) Representative images of hM3Dq-mCherry expression in the adBNST (left) and AAV2 injection site visualized by coinjection of fluorescent beads in the LH (right). Scale bars, 300 μm. (**C**) Schedule of experiments. (**D** to **G**) Behavioral analyses using OFT (D), EPM (E), and LDB (F) tests, and emotionality *z* score across these tests (G). Box-whisker plots show the median, interquartile range, and 10th to 90th percentiles. Statistical significance was evaluated using two-way ANOVA followed by Bonferroni’s post hoc test (*n* = 9 for each group). *P < 0.05 and **P < 0.01. (**H**) Mechanical thresholds were assessed using the von Frey test in mCherry-expressing nerve injury mice (*n* = 9) and hM3Dq-mCherry–expressing nerve injury mice (*n* = 9) before and after CNO administration (1 mg/kg, intraperitoneally). Statistical significance was evaluated using two-way repeated-measures ANOVA followed by Bonferroni’s post hoc test. Details of statistical data are provided in table S1.

To examine whether the activation of LH-projecting adBNST neurons ameliorated chronic pain–induced anxiety-like behavior, we bilaterally injected the retrograde-transported AAV2-expressing Cre recombinase into the LH, and AAV5 Cre-dependently expressing hM3Dq-mCherry into the adBNST (Fig. 4, A and B). We also infused the same AAV2 into the LH and AAV5 carrying a Cre-dependent mCherry alone construct into the adBNST as controls. The mice underwent surgery for chronic pain more than 10 days after the injections (Fig. 4C). Histological analyses showed that intraperitoneal administration of CNO (1 mg/kg) increased c-Fos expression in hM3Dq-mCherry–positive adBNST neurons (fig. S8, A to C), indicating that CNO administration activated LH-projecting adBNST neurons in vivo. Behavioral analyses were performed 4 weeks after surgery for neuropathic pain. In the comparison between the sham-mCherry and nerve injury–mCherry groups, significant differences were detected in the LDB test and emotionality *z* score. Time spent in the open arms in the EPM (Fig. 4E) and time spent in the light side in the LDB (Fig. 4F) were longer in the nerve injury–hM3Dq group compared with the nerve injury–mCherry group, indicating amelioration of anxiety-like behavior by the activation of LH-projecting adBNST neurons. Emotionality *z* score also showed a significant amelioration of anxiety-like behavior in the hM3Dq-mCherry group (Fig. 4G and fig. S9, B to D). Chemogenetic activation of LH-projecting adBNST neurons showed no significant effects on anxiety-like behavior in sham-operated mice (Fig. 4, D to G), while Kim *et al.* (*16*) reported that optogenetic activation of the adBNST-LH pathway produced an anxiolytic effect in naïve mice. It is likely that the experimental conditions of chemogenetic activation used in this study were not sufficient to produce anxiolytic effects in normal (nonchronic pain) mice. Activation of LH-projecting adBNST neurons tended to ameliorate mechanical allodynia in the von Frey test (Fig. 4H), although the difference was not significant. These results suggest that attenuated activity of LH-projecting adBNST neurons plays a critical role in chronic pain–induced anxiety-like behavior.

### Oval BNST^CART^ neurons send inhibitory inputs to LH-projecting adBNST neurons

**Fig. 5.**
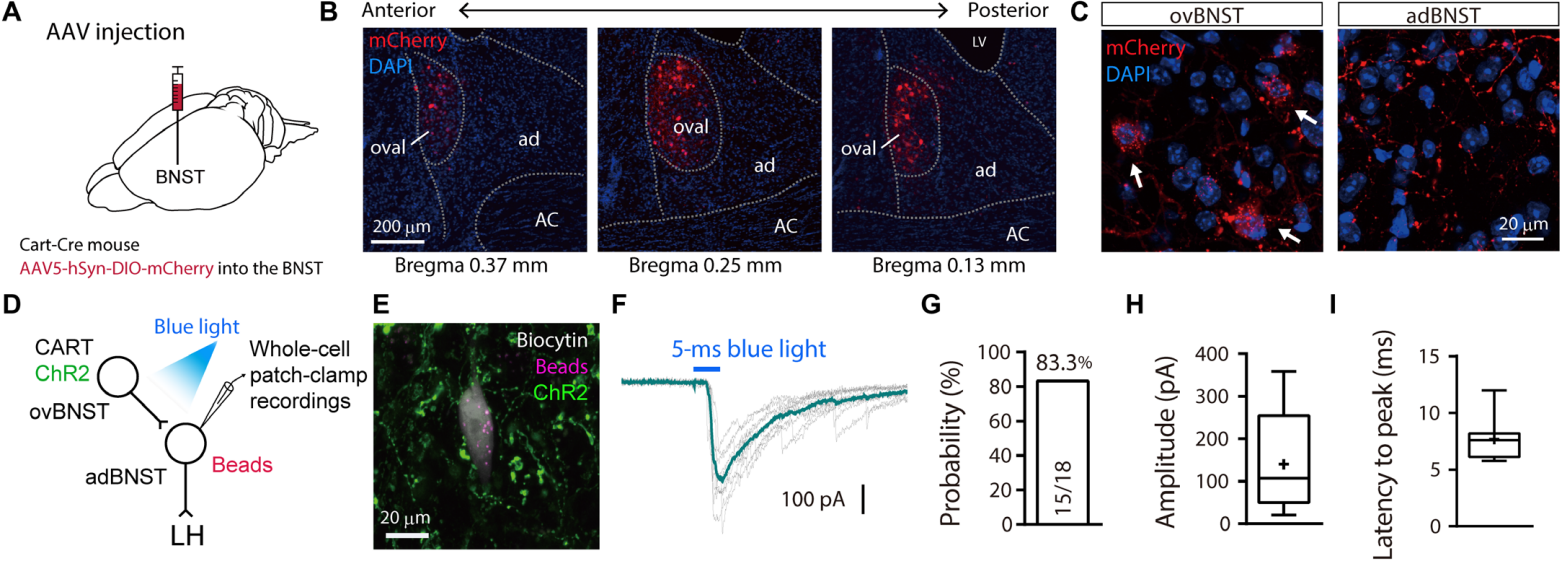
LH-projecting adBNST neurons receive inhibitory inputs from CART-expressing BNST neurons. (**A**) Scheme of experiments. Cart-Cre mice were injected with AAV5 delivering a Cre-dependent mCherry expression construct to visualize CART-positive BNST neurons. (**B**) Confocal images of coronal brain sections showing mCherry-expressing neurons (red) in the anterior BNST. Approximate distances (anterior-posterior) from the bregma are shown. ac, anterior commissure; ad, adBNST; ov, ovBNST. Scale bar, 200 μm. DAPI, 4′,6-diamidino-2-phenylindole. (**C**) High-magnification images of the ovBNST (left) and adBNST (right). Arrows indicate mCherry expression in soma. Scale bar, 20 μm. (**D**) Scheme of experiments. AAV5 delivering Cre-dependent ChR2 expression construct was injected into the ovBNST of Cart-Cre mouse and then the red retrobeads were injected into the LH. Whole-cell patch-clamp recordings from retrobead-labeled adBNST neurons were performed to examine the electrophysiological responses to blue light stimulation. (**E**) Confocal image of a retrobead-labeled BNST neuron filled with biocytin during recording. Scale bar, 20 μm. (**F**) Representative trace showing light-evoked IPSCs. (**G**) Synaptic connectivity from ovBNST^CART^ neurons to *I*_h_-negative LH-projecting adBNST neurons. Fifteen out of 18 cells from two mice showed light-evoked IPSCs. (**H** and **I**) Summary of amplitude (H) and latency to peak (I) in recorded neurons (*n* = 15 cells from two mice). Box-whisker plots indicate median, interquartile range, and 10th to 90th percentiles. Means are indicated by “+” in (H) and (I).

Since most BNST neurons are GABAergic (*17*), it is possible that the excitability of inhibitory interneurons within the BNST was enhanced by chronic pain, thereby increasing inhibitory inputs to the downstream BNST output neurons. To test this, transgenic mice that expressed Cre recombinase in cocaine- and amphetamine-regulated transcript (CART)–positive neurons (Cart-Cre mice) were used to manipulate a specific subpopulation of BNST neurons because this peptide specifically expresses in the BNST subnuclei (*20*, *21*). Histological analyses following injections of AAV5-hSyn-DIO-mCherry into the BNST of Cart-Cre mice revealed that CART-positive BNST neurons were specifically localized in the oval region (ovBNST) within the anterior BNST (Fig. 5, A to C). CART-positive axon fibers visualized by mCherry were observed in the adBNST (Fig. 5C). Double immunostaining revealed that most (>80%) CART-positive neurons were protein kinase Cδ (PKCδ) positive, while about 30% of PKCδ-positive neurons were CART positive (fig. S10).

To examine functional synaptic connectivity from ovBNST^CART^ neurons to *I*_h_-negative LH-projecting adBNST neurons, electrophysiological analyses were conducted using BNST slices prepared from Cart-Cre mice injected with retrobeads into the LH and AAV5-DIO-hSyn-ChR2-EYFP into the ovBNST (Fig. 5, D and E). Whole-cell patch-clamp recordings detected light-evoked IPSCs in 83.3% of the recorded LH-projecting adBNST neurons (15 of 18 neurons; Fig. 5, F to I), suggesting that ovBNST^CART^ neurons make inhibitory synapses onto LH-projecting adBNST neurons.

To determine whether ovBNST^CART^ neurons make inhibitory synapses onto other output neurons regulating anxiety-like behavior, electrophysiological analyses were performed using BNST slices prepared from Cart-Cre mice injected with AAV9-DIO-hSyn-ChR2-EYFP into the ovBNST and retrobeads into the VTA (fig. S11A). Whole-cell patch-clamp recordings detected light-evoked IPSCs in 57.9% of the recorded VTA-projecting adBNST neurons (11 of 19 neurons; fig. S11, B to E), indicating that ovBNST^CART^ neurons also make inhibitory synapses onto VTA-projecting adBNST neurons. Histological analysis using red and green retrobeads, which were injected into the LH and VTA, respectively, showed that approximately 25% of adBNST neurons had projections to the LH and VTA, and about half of these neurons sent their projections to both brain regions (fig. S11, F to H). We also examined the overlap between the LH-projecting and VTA-projecting neurons by injecting the retrograde-transported AAV2-expressing Cre recombinase into the LH and AAV9-CBA-FLEX-Synaptophysin-mCherry into the adBNST to visualize the nerve terminals of LH-projecting adBNST neurons (fig. S11, I to N). Synaptophysin-mCherry–positive puncta were observed not only in the LH but also in the VTA.

### Chronic pain–induced changes in intrinsic neuronal states of ovBNST^CART^ neurons

**Fig. 6.**
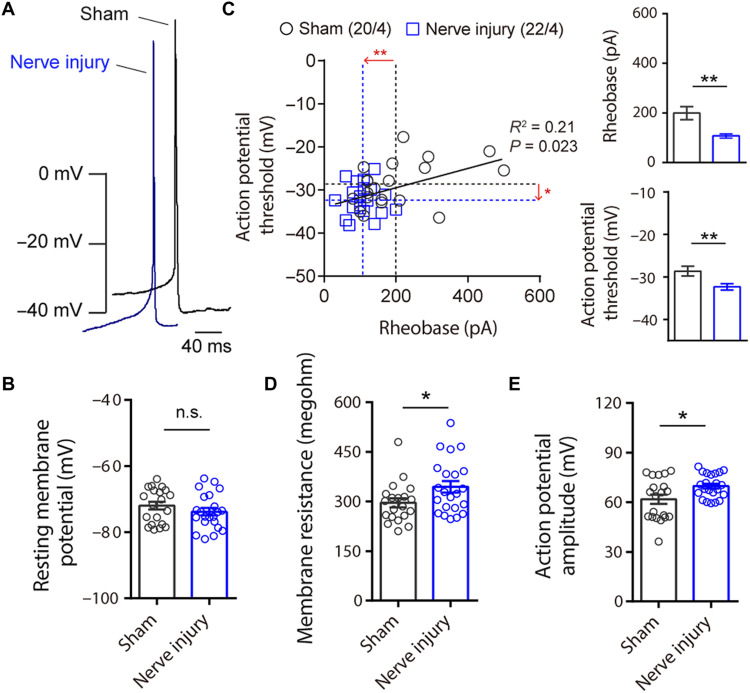
Chronic pain enhances the excitability of ovBNST^CART^ neurons. (**A**) Representative action potentials in ovBNST^CART^ neurons of sham (black) and nerve-injured (blue) mice. (**B** to **E**) Intrinsic electrophysiological properties of ovBNST^CART^ neurons of sham (black, *n* = 20 cells from four mice) and nerve injury (blue, *n* = 22 cells from four mice) mice: resting membrane potential (B), relationship between action potential threshold and rheobase (C), membrane resistance (D), and action potential amplitude (E). Data are expressed as means ± SEM. Statistical significance was evaluated using two-tailed unpaired *t* test. *P < 0.05 and **P < 0.01. Details of statistical data are provided in table S1.

To determine whether chronic pain changes the physiological properties of ovBNST^CART^ neurons, we performed whole-cell patch-clamp recordings from ovBNST^CART^ neurons visualized by injecting AAV5-hSyn-DIO-mCherry into the BNST of Cart-Cre mice (Fig. 6A). Although the resting membrane potential was indistinguishable between the sham and nerve injury mice (Fig. 6B), significant reductions in action potential threshold and rheobase were observed in the nerve injury group (Fig. 6C). Consistent with the decrease in rheobase, membrane resistance increased in the nerve injury mice (Fig. 6D). In addition, the action potential amplitude in the nerve injury mice was higher than that in the sham mice (Fig. 6E). These results demonstrated that chronic pain elevated ovBNST^CART^ neuron excitability.

### Elevated excitability of ovBNST^CART^ neurons contributes to increased inhibitory inputs to LH-projecting adBNST neurons

**Fig. 7.**
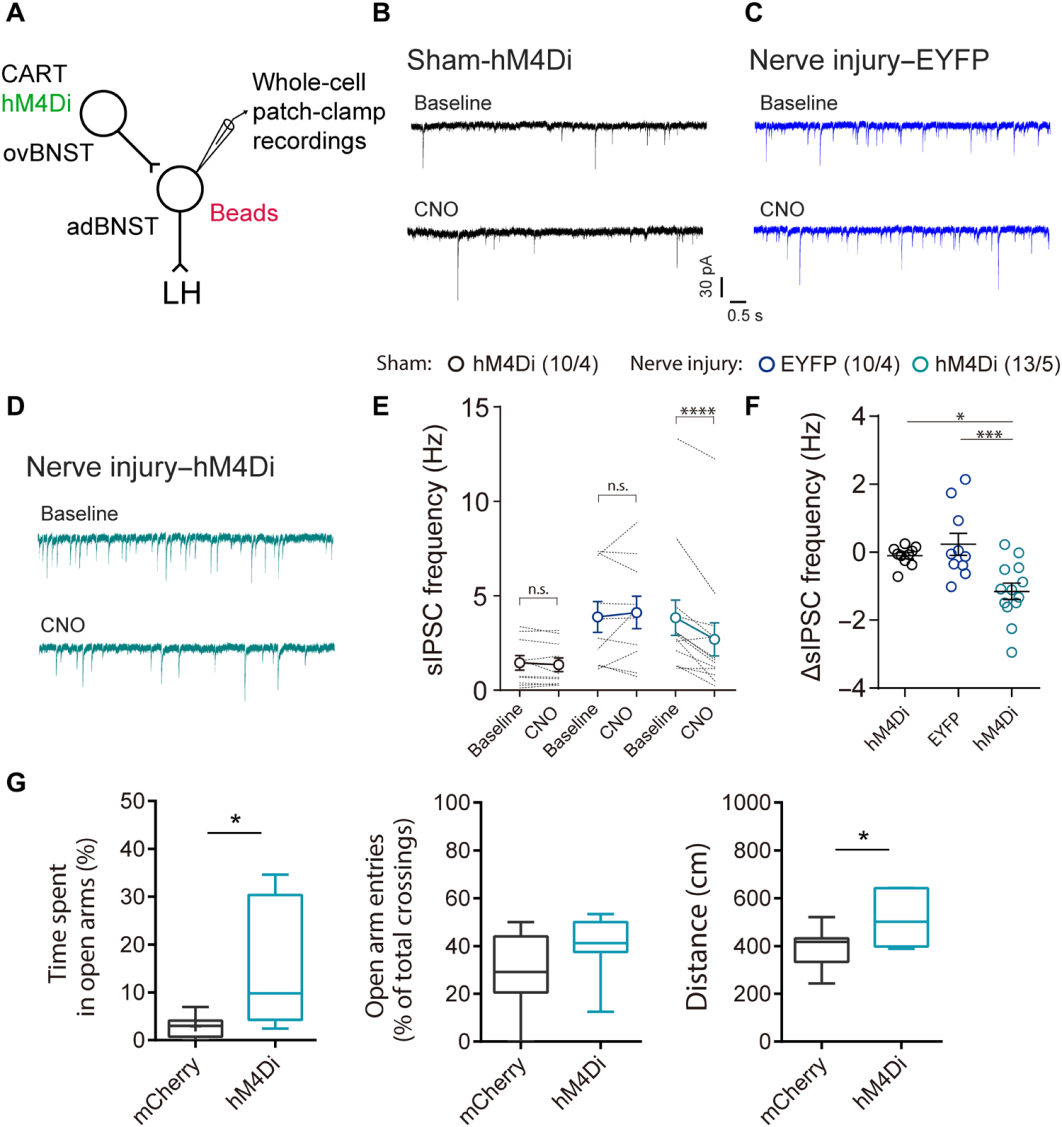
Involvement of enhanced excitability of ovBNST^CART^ neurons in increased inhibitory inputs to LH-projecting adBNST neurons and chronic pain–induced anxiety-like behavior. (**A**) Scheme of experiments. (**B** to **D**) Representative traces of sIPSCs in LH-projecting adBNST neurons before and after CNO (10 μM) bath application in sham mice that expressed hM4Di (B; *n* = 10 from four mice), nerve injury mice that expressed EYFP alone (C; *n* = 10 from four mice), and nerve injury mice that expressed hM4Di (D; *n* = 13 from five mice). (**E** and **F**) Effect of CNO bath application on sIPSC frequency. Data are expressed as means ± SEM. Statistical significance was evaluated using two-way repeated-measures ANOVA followed by Bonferroni’s post hoc test (E) and one-way ANOVA followed by Sidak’s post hoc test (F). *P < 0.05; ***P < 0.01; ****P < 0.001. (**G**) Behavioral analyses using an EPM test (mCherry: *n* = 8; hM4Di: *n* = 7). Box-whisker plots indicate median, interquartile range, and 10th to 90th percentiles. Statistical significance was evaluated using two-tailed unpaired Student’s *t* test. *P < 0.05. Details of statistical data are provided in table S1.

To assess the role of the elevated excitability of ovBNST^CART^ neurons in increased inhibitory synaptic inputs to LH-projecting adBNST neurons, the neuronal activity of ovBNST^CART^ neurons was manipulated using a chemogenetic technique (Fig. 7A). BNST slices were prepared from Cart-Cre mice injected with AAV vectors that Cre-dependently express hM4Di-mCitrine or enhanced yellow fluorescent protein (EYFP) alone into the BNST, and then injected with retrobeads into the LH. Whole-cell patch-clamp recordings from *I*_h_-negative LH-projecting adBNST neurons revealed that the bath application of CNO (10 μM) significantly decreased the frequency of sIPSCs in the nerve injury mice, which was elevated by chronic pain, but not less than the sIPSC frequency in the sham-operated mice (Fig. 7, B to F). No changes were observed in the nerve injury mice expressing EYFP alone. Chemogenetic inhibition of ovBNST^CART^ neurons did not affect the sIPSC frequency in LH-projecting adBNST neurons of sham-operated mice. These results suggest that the elevated excitability of ovBNST^CART^ neurons plays an important role in the increased inhibitory inputs to LH-projecting adBNST neurons in a mouse model of chronic pain.

### Elevated excitability of ovBNST^CART^ neurons plays a critical role in chronic pain–induced maladaptive anxiety

To examine the involvement of elevated excitability of ovBNST^CART^ neurons in chronic pain–induced maladaptive anxiety, we performed the EPM test using a mouse model of chronic pain, prepared from Cart-Cre mice injected with AAV5-hSyn-DIO-hM4Di-mCherry or AAV5-hSyn-DIO-mCherry into the ovBNST. Inhibition of ovBNST^CART^ neurons by CNO (1 mg/kg) administration significantly increased the time spent in open arms in the nerve injury mice expressing hM4Di compared to the control group expressing mCherry alone (Fig. 7G).

## DISCUSSION

The comorbidity of chronic pain and psychiatric disorders, such as depression and anxiety disorders, has long been recognized clinically (*2*–*4*), and preclinical studies have reported depression- and anxiety-like behaviors in animal models of chronic pain (*5*–*8*). These findings suggest a common neuronal basis for these pathological states. However, neuronal mechanisms of chronic pain–induced depression/anxiety-like behaviors remain to be elucidated. The present study demonstrated that (i) chronic pain increased inhibitory synaptic inputs to LH-projecting adBNST neurons; (ii) chemogenetic inhibition of LH-projecting adBNST neurons induced anxiety-like behavior in naïve mice; and (iii) chemogenetic activation of LH-projecting adBNST neurons ameliorated anxiety-like behavior in a mouse model of chronic pain. These results suggest that sustained inhibition of LH-projecting adBNST neurons plays a critical role in chronic pain–induced anxiety-like behavior. We have previously reported that inhibitory inputs to VTA-projecting BNST neurons increased not only in a rat model of chronic pain (*15*) but also in rats exposed to chronic mild stress, an animal model of depression (*22*). Recently, Pati *et al.* (*23*) reported that the frequency of sIPSCs in VTA/LH-projecting BNST neurons was increased in an animal model of alcohol withdrawal. These findings suggest that increased inhibitory inputs to BNST neurons projecting to other brain regions, such as the LH and VTA, may be a common neuronal mechanism underlying emotional dysfunctions, such as anxiety and depression, induced by chronic pain, chronic stress, and alcohol dependence.

BNST neurons have been characterized according to their projection targets in previous studies. Activation of LH-projecting BNST neurons suppresses anxiety-like behavior in the EPM test, while activation of BNST neurons projecting to the lateral parabrachial nucleus and VTA causes decreased respiratory rates and place preference, respectively (*16*). Recently, Giardino *et al.* (*24*) reported that LH-projecting BNST neurons can be further classified into two subpopulations that express corticotropin-releasing factor (CRF) and cholecystokinin. These two subpopulations of LH-projecting BNST neurons specifically respond to rewarding and aversive stimuli, respectively, and drive opposing emotional behaviors. These findings suggest that LH-projecting BNST neurons are heterogeneous in terms of the gene expression patterns and neural circuit formations. In this study, we demonstrated that LH-projecting adBNST neurons can be classified into two different cell types: *I*_h_ negative and *I*_h_ positive. Chronic pain increased inhibitory inputs to *I*_h_-negative neurons, but not to *I*_h_-positive neurons, suggesting that distinct neuronal circuits regulate their activity. To elucidate the role of these two types of LH-projecting adBNST neurons in the regulation of emotional states, further studies are required to identify the molecular markers of these neurons for selective recording and manipulation.

Most BNST neurons are GABAergic (*17*), and some of them make inhibitory synapses within the BNST (*25*, *26*). The neural circuitry in the lateral part of the central amygdala (CeL) has been well studied as an intranuclear reciprocal inhibitory circuit between genetically distinct neuronal populations, i.e., PKCδ-positive and PKCδ-negative neurons. PKCδ-negative neurons are represented by somatostatin- or CRF-positive neurons, and it has been reported that these three cell types cover the majority of CeL neurons and regulate fear and anxiety behaviors (*27*–*31*). A recent study using immunostaining for PKCδ combined with genetic labeling for CRF or somatostatin neurons showed similar compositions of these three major cell types between the CeL and the ovBNST (*32*), suggesting that a similar intranuclear reciprocal inhibitory circuit that regulates fear/anxiety behaviors also exists in the ovBNST. It was reported that inhibition of PKCδ-positive neurons in the CeL (*31*) and the ovBNST (*32*) increased the time in open arms in the EPM test, suggesting that PKCδ-positive neurons in these brain regions play a crucial role in anxiety-like behavior. However, since PKCδ-positive neurons make up a fairly large proportion (35 to 40%) of the ovBNST neurons (*32*), it may be necessary to use specific cellular markers expressed in a limited number of ovBNST neurons to determine which population of neurons are involved in the regulation of negative emotions, such as anxiety and fear. In this study, we used Cart-Cre mice. CART is expressed specifically in the oval region within the anterior BNST (Fig. 5B). More than 80% of CART-positive neurons are PKCδ positive, while about 30% of PKCδ-positive neurons are CART positive (fig. S10). Recently, Walker *et al.* (*33*) reported a similar colocalization pattern of PKCδ and CART in rat central amygdala (CeA) (97% of CART-positive neurons were PKCδ positive, while 34% of PKCδ-positive neurons were CART positive). These findings indicated that, rather than PKCδ-Cre mice, Cart-Cre mice could be used to manipulate a more restricted population of ovBNST neurons.

**Fig. 8.**
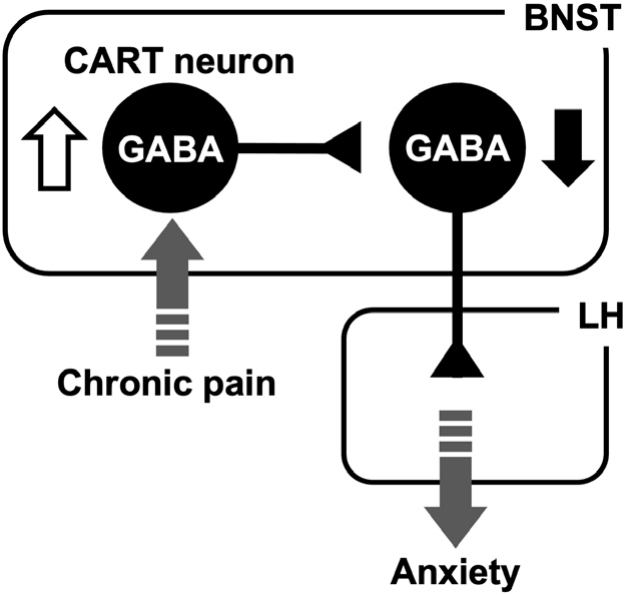
Neuronal circuit involved in chronic pain–induced maladaptive anxiety. Increased excitability (white arrow) of ovBNST^CART^ neurons causes a sustained suppression (black arrow) of LH-projecting adBNST neurons during chronic pain, thereby enhancing anxiety-like behavior.

In this study using Cart-Cre mice, we found that (i) ovBNST^CART^ neurons make inhibitory synapses onto LH-projecting adBNST neurons; (ii) chronic pain elevated the excitability of ovBNST^CART^ neurons; (iii) chemogenetic inhibition of ovBNST^CART^ neurons decreased the inhibitory inputs to LH-projecting adBNST neurons in brain slices prepared from a mouse model of chronic pain; and (iv) chemogenetic inhibition of ovBNST^CART^ neurons ameliorated chronic pain–induced anxiety-like behavior. These results suggest that the increased excitability of ovBNST^CART^ neurons causes a sustained suppression of LH-projecting adBNST neurons during chronic pain, thereby enhancing anxiety-like behavior (Fig. 8). Recently, Rodriguez-Romaguera *et al.* (*34*) demonstrated that activation of prepronociceptin (Pnoc)–expressing BNST neurons induced anxiety-like behavior in an EPM test. They showed that 88% of Pnoc-expressing BNST neurons were distributed among 4 of the 11 neuronal clusters identified using a single-cell sequencing technique and that two of these four clusters expressed CART prepropeptide transcript. Increased inhibitory inputs to LH-projecting BNST neurons may be involved in the anxiety-like behavior induced by activation of Pnoc-expressing BNST neurons.

To determine whether ovBNST^CART^ neurons make inhibitory synapses onto other output neurons regulating anxiety-like behavior, we examined the effect of optogenetic stimulation of ovBNST^CART^ neurons on the inhibitory inputs recorded from VTA-projecting BNST neurons, the activation of which was reported to exert an anxiolytic effect in the EPM test (*35*). Patch-clamp electrophysiology revealed that optogenetic stimulation of ovBNST^CART^ neurons evoked IPSCs in VTA-projecting adBNST neurons and in LH-projecting adBNST neurons. Furthermore, histological analyses demonstrated an overlap between LH-projecting and VTA-projecting adBNST neurons as reported by Marcinkiewcz *et al.* (*26*). These results suggest that chemogenetic inhibition of ovBNST^CART^ neurons reduces inhibitory inputs not only to LH-projecting adBNST neurons but also to VTA-projecting adBNST neurons. The reduction of inhibitory inputs to VTA-projecting adBNST neurons may contribute to the anxiolytic effect of chemogenetic suppression of ovBNST^CART^ neurons in a mouse model of chronic pain.

Intracerebroventricular injections of CART have been reported to induce anxiety-like behavior in the EPM test (*36*). To our knowledge, however, the role of CART within the BNST in the regulation of negative emotions, such as anxiety and fear, has not yet been investigated. As for the CeA, Rale *et al.* (*37*) reported that the fear response induced by trimethylthiazoline (a fear-inducing odorant derived from fox dung) was enhanced and attenuated by intra-CeA injection of CART and anti-CART neutralizing antibodies, respectively. Furthermore, Walker *et al.* (*33*) reported that intra-CeA injection of anti-CART neutralizing antibodies attenuated yohimbine-induced anxiety-like behavior in the LDB test during alcohol abstinence. Given the anatomical and functional similarities between CeA and BNST (*32*), these findings suggest that CART within the BNST may also play a crucial role in the regulation of negative emotions, such as anxiety and fear. Further electrophysiological and behavioral studies are needed to clarify the effects of CART itself, rather than CART-expressing neurons, on the excitability of LH-projecting BNST neurons and anxiety-like behavior.

In conclusion, electrophysiological and behavioral analyses using chemogenetic manipulation of neuronal activity demonstrated that chronic pain increased inhibitory synaptic inputs to LH-projecting adBNST neurons, which in turn induced maladaptive anxiety. Further investigations using Cart-Cre mice to manipulate specific subpopulation of BNST neurons demonstrated that elevated excitability of ovBNST^CART^ neurons caused the increased inhibitory inputs to LH-projecting adBNST neurons during chronic pain, leading to enhanced anxiety-like behavior. These findings demonstrate how chronic pain–induced neuroplastic changes induce psychiatric disorders characterized by maladaptive anxiety.

## MATERIALS AND METHODS

### Animals

Male 8- to 10-week-old C57BL/6JJmsSlc (Japan SLC, Shizuoka, Japan) and Tg(Cartpt-cre) 1Aibs/J (Cart-cre, Jackson Laboratory, Bar Harbor, ME) mice were used in this study. Mice were kept in a constant ambient temperature (23° ± 1°C) with ad libitum access to food and water in a 12-/12-hour light/dark cycle, with the day starting at 7:00 a.m. The mice were randomly assigned to groups. Behavioral experiments were conducted during the light cycle. All experiments were approved by the Institutional Animal Care and Use Committee of Hokkaido University.

### Virus vectors

AAV2-retro/CAG-Cre, AAV5- DIO-EF1α-ChR2-EYFP, and AAV5-EF1α-DIO-EYFP were purchased from the University of North Carolina Vector Core (Chapel Hill, NC, USA). AAV5-hSyn-DIO-hM4Di-mCherry (44362-AAV5), AAV5-hSyn-DIO-hM3Dq-mCherry (44361-AAV5), AAV5-hSyn-DIO-mCherry (50459-AAV5), AAV8-hSyn-DIO-HA-hM4Di-IRES-mCitrine (44361-AAV8), and AAV9-EF1a-double floxed-hChR2(H134R)-EYFP-WPRE-HGHpA were purchased from Addgene (Watertown, MA, USA). AAV9-CBA-FLEX-Synaptophysin-mCherry was gifted by A. Uematsu and J. P. Johansen (*38*).

### Intracranial injections

The mice were anesthetized with isoflurane (induction, 3.0%; maintenance, 1.0 to 1.5%) in oxygen-enriched air and fixed in a stereotaxic frame (SR-6 M-HR; Narishige, Tokyo, Japan). Lidocaine was topically applied to the scalp to alleviate pain before the incision. The virus solution was injected into the adBNST [anterior-posterior (AP), +0.5 mm; medial-lateral (ML), 1.1 mm; and dorsal-ventral (DV), 4.0 mm to bregma], ovBNST (AP: +0.6 mm; ML, 1.2 mm; and DV, 3.9 mm to bregma), or LH (AP, −1.1 mm; ML, 1.2 mm; and DV, 5.6 mm to bregma) in a volume of 250 nl per side using a 30-gauge cannula connected to a syringe pump (Legato 101, KD Scientific, Holliston, MA, USA). Following the injection, the hole in the skull was sealed with dental cement, and the scalp incision was sutured. After the surgery, meloxicam (1 mg/kg) was injected subcutaneously to alleviate postoperative pain. To identify the AAV2-retro/CAG-Cre injection site, green fluorescent microspheres (Lumafluor Corp., Carlisle, NC, USA) were added to the virus solution at a volume ratio of 1:1000. To visualize LH-projecting BNST neurons, 300 nl of red fluorescent microspheres (red retrobeads, Lumafluor) diluted with saline (1:2) was unilaterally injected into the LH.

### Surgery for the chronic pain model

The spared nerve injury model was used as an animal model of chronic pain (*39*). Mice (8 to 10 weeks old) were anesthetized using isoflurane (induction, 3.0%; maintenance, 2.0%). An incision was made on the left thigh, and the underlying muscles were separated via blunt resection to expose the three branches of the sciatic nerve bundle. The tibial and the common peroneal nerves were tightly ligated using 5-0 silk sutures and then completely transected distal to the ligation sites, leaving the sural nerve intact. Lidocaine was topically applied to the incision site. The overlying muscle and skin were sutured in layers. Postoperatively, 0.1% gentamicin ointment was applied topically, and the mice were kept in a warming chamber for 30 min. Sham surgery included exposure of the sciatic nerve, but the tibial and common peroneal nerves were not injured. Postoperatively, the mice were housed individually.

The von Frey test was performed to assess tactile allodynia. The mice were placed on the elevated wire grids for at least 20 min for habituation before the test. A series of von Frey monofilaments calibrated at 0.04, 0.07, 0.16, 0.40, 0.60, 1.00, and 2.00 g (North Coast Medical, Inc., Morgan Hill, CA, USA) were used to stimulate the plantar surface of the hind paw, starting with the 0.16-g filament. The 50% paw withdrawal threshold was determined using the up-down method (*40*).

### Behavioral tests

The mice were habituated to a testing room for at least 30 min on each test day. Behavioral tests were conducted over 3 days in the order of OFT, EPM, and LDB tests. In the experiments using a designer receptors exclusively activated by designer drugs (DREADD) technique, CNO (1.0 mg/kg; Abcam, Cambridge, UK) dissolved in saline was injected intraperitoneally 40 min before the start of each behavioral test. The Ethovision XT video tracking system (Noldus, Wageningen, Netherlands) was used to analyze the animal behaviors.

#### Open field test

An open-topped white box (width, 46 cm; depth, 46 cm; and height, 23 cm), in which the light intensity was 50 to 60 l×, was placed in a sound-attenuated box. The area was virtually divided into central (23 × 23 cm) and peripheral areas. At the start of the test, each animal was placed in the peripheral area. The movement of the animal body center was tracked for 5 min, and the distance traveled, the time spent in the center area, and the number of times the center area was entered were calculated.

#### EPM test

The apparatus was composed of two closed arms (30 cm each), two open arms (30 cm each), and a central area (7 × 7 cm). It was elevated 40 cm above the floor and illuminated using white dim light (6 ± 1 l× at the central area and open arms and 3 ± 1 l× at the closed arms). At the start of the test, each animal was placed in the central area. The movement of the animal was tracked for 5 min using a camera placed above the apparatus, and the time spent in the open arms, the number of times the open arm was entered, and the distance traveled were measured.

#### LDB test

The apparatus, consisting of two connected chambers [an open-topped 32 × 25 × 25–cm white box (light side: 470 to 500 l×) and a close-topped 16 × 25 × 25–cm black box (dark side) with a 7 × 7–cm connecting doorway] was placed in a sound-attenuated box. At the start of the test, each animal was placed in the dark box, and its movement in the light side was tracked for 5 min. The time spent in the light side, the latency to the first entry to the light side, and the number of transitions between the two sides were measured.

#### Emotionality z score

The *z* scores were calculated using the following formula: *z* = (*X* − μ)/σ (*41*). *X* represents the individual data for the observed parameter, while μ and σ represent the mean and the standard deviation for the control group, respectively. Behavioral parameters related to emotional aspects (time spent in the center zone in the OFT, time spent in the open arms in the EPM, and time spent in the light side in the LDB) were used to calculate the *z* score for each animal in each test. The emotionality *z* score for each animal was then calculated as the average of the *z* scores in the OFT, EPM, and LDB tests.

#### NSF test

After 24 hours of food deprivation, the mice were habituated for 1 hour in the testing room. An open-topped white box (width, 46 cm; depth, 46 cm; and height, 23 cm) in which the light intensity was 1000 to 1050 l×, was used. After putting a food pellet crushed into 1.0 to 2.0 g at the center of the arena, the mice were placed at the corner of the arena. Latency to feed was measured as the amount of time elapsed before taking a bite of the food pellet. After the NSF test, the mice were placed in the cage with a preweighed food pellet. After 30 min, the food pellet was reweighed to determine home cage consumption.

### Slice preparation for electrophysiological recordings

Artificial cerebrospinal fluid (ACSF; 126 mM NaCl, 2.5 mM KCl, 1.25 mM NaH_2_PO_4_, 1 mM MgCl_2_, 2 mM CaCl_2_, 26 mM NaHCO_3_, and 10 mM glucose, oxygenated with 95% O_2_/5% CO_2_ at pH 7.3) and a cutting solution containing choline chloride (113.9 mM) instead of NaCl were prepared. Mice were deeply anesthetized using pentobarbital (100 mg/kg, intraperitoneally) and transcardially perfused with ice-cold cutting solution. The brain was then removed and cut into 250-μm-thick coronal sections in the ice-cold cutting solution using a vibratome (VT1200S, Leica Microsystems GmbH, Wetzlar, Germany). Brain slices were kept in ACSF at 32° to 34°C for 25 to 30 min and then at room temperature until the electrophysiological recordings.

### Electrophysiological recordings

The slices were transferred to a recording chamber on an upright microscope (BX50WI; Olympus, Tokyo, Japan) and constantly perfused with ACSF saturated with 95% O_2_/5% CO_2_ at 32° to 34°C at a perfusion rate of 1.0 to 1.5 ml/min. The neurons in the brain slices were visualized using an infrared (IR) camera (IR-1000, Dage-MTI, Michigan City, IN, USA). Projection neurons labeled with retrobeads or neurons expressing fluorescent proteins were visualized using epifluorescence and a 40× objective (LUMPlanLF N 40×/0.80; Olympus). Current clamp recordings were performed to classify the BNST neurons before the recordings of synaptic currents or measurement of membrane potentials and input resistance. To monitor the responses of BNST neurons to hyperpolarizing current injections, an initial membrane potential of −60 mV was used, followed by a series of current injections (−20 pA steps, 400 ms in duration) ranging from 0 to −180 pA. The *I*_h_ current was identified as described previously (*19*). Specifically, the occurrence of *I*_h_ was verified by a “voltage-sag” equal to or greater than 10% of the total voltage deflection upon the hyperpolarizing current injection, which elicited a membrane voltage of approximately −100 mV. All the recorded neurons were classified as either *I*_h_ positive or *I*_h_ negative.

#### Measuring synaptic events

Glass electrodes were pulled from thin-walled borosilicate glass capillaries using a micropipette puller (Model P-1000IVF, Sutter Instrument, Novato, CA, USA). To record the synaptic currents, we used recording glass electrodes with 3- to 7-megohm tip resistance when filled with an internal solution [65 mM K-gluconate, 70 mM KCl, 10 mM Hepes, 0.5 mM EGTA, 1 mM MgCl_2,_ 12 mM Na-phosphocreatine, 4 mM Mg–adenosine triphosphate (ATP), 0.5 mM Na–guanosine triphosphate (GTP), and 0.2% biocytin, adjusted to pH 7.2 to 7.3 with KOH]. Voltage-clamp recordings were performed at a holding potential of −70 mV. To measure sIPSCs, 6-cyano-7-nitroquinoxaline-2,3-dione (CNQX; 10 μM; Sigma-Aldrich, St. Louis, MO, USA) and 2-amino-5-phosphonopetanoic-acid (AP5; 50 μM; Abcam) were added to ACSF to exclude EPSCs. To measure mIPSCs, CNQX (10 μM), AP5 (50 μM), and tetrodotoxin (1 μM; Wako, Osaka, Japan) were added to ACSF to exclude EPSCs and IPSCs caused by network activity. To measure sEPSCs, picrotoxin (100 μM; Sigma-Aldrich) was added to ACSF to exclude IPSCs via GABA_A_ (γ-aminobutyric acid type A) receptors. Synaptic currents were recorded for 1 min, and 20 recordings from each cell were used to determine the cumulative probability.

#### Chemogenetics

To validate the hM4Di function, current-clamp recordings were conducted at a resting membrane potential from hM4Di-mCherry–expressing adBNST neurons using glass pipettes with 3- to 7-megohm tip resistance when filled with an internal solution (132 mM K-gluconate, 3 mM KCl, 10 mM Hepes, 0.5 mM EGTA, 1 mM MgCl_2_, 12 mM Na-phosphocreatine, 4 mM Mg-ATP, 0.5 mM Na-GTP, and 0.2% biocytin, adjusted to pH 7.2 to 7.3 with KOH). To monitor the changes in input resistance, a −20-pA current was injected every 20 s. After confirming the stable baseline recordings, CNO (10 μM) was bath applied for 5 min, and the membrane potential and input resistance were compared between prior to (−1 to 0 min) and following (4 to 5 min) CNO application. To measure sIPSC changes in *I*_h_-negative LH-projecting adBNST neurons, CNO (10 μM) was bath applied for 5 min, and the frequency and amplitude of sIPSCs were compared between prior to (−1 to 0 min) and following (4 to 5 min) CNO application.

#### Optogenetics

To confirm functional synaptic connectivity, blue light stimulations (λ = 465 nm, 5-ms duration; LEX2-LZ4-B; Brainvision Inc., Tokyo, Japan) were delivered to the BNST slices containing ChR2-expressing neurons, and light-evoked IPSCs were recorded from LH-projecting adBNST neurons labeled with red retrobeads. Functional synaptic connectivity was defined to be present when evoked IPSCs with an amplitude of 10 pA or greater were observed in at least 60% of the light stimulation trials (6 of 10 stimulations).

#### Measuring intrinsic physiological properties

Intrinsic physiological properties of mCherry-expressing neurons were analyzed using 3- to 7-megohm glass pipettes filled with an internal solution (132 mM K-gluconate, 3 mM KCl, 10 mM Hepes, 0.5 mM EGTA, 1 mM MgCl_2_, 12 mM Na-phosphocreatine, 4 mM Mg-ATP, 0.5 mM Na-GTP, and 0.2% biocytin, pH 7.2 to 7.3 adjusted with KOH). The procedure was as follows: (i) Resting membrane potential was measured in a current-clamp mode without current injection for 60 s; (ii) membrane resistance was measured by injecting −20-pA step current (5-s duration) from 0 to −80 pA with the membrane potential held at −60 mV; (iii) action potential threshold, action potential amplitude, and rheobase were measured by injecting +10 pA step current (5-s duration). The action potential threshold was defined as the membrane potential at which the derivative of the voltage (*dV*/*dt*) exceeded 10 mV/ms.

All data were acquired using a Multiclamp 700B amplifier, filtered at 0.2 kHz, digitized at 10 kHz, and analyzed using the pClamp10 software (Molecular Devices, Sunnyvale, CA, USA) and Matlab (Mathworks Inc., Natick, MA, USA). Data from the neurons with resting membrane potential more positive to −50 mV or in which the action potential did not overshoot were excluded from the statistical analyses. Access resistance was monitored by injecting a current (−5 mV and 50 ms) every 20 s. Data from the neurons with access resistance greater than 30 megohms or that changed by 20% or more during the recordings were also excluded.

### Histology

The mice were deeply anesthetized using pentobarbital (100 mg/kg, intraperitoneally) and then transcardially perfused with phosphate-buffered saline (PBS), followed by 4% paraformaldehyde (PFA) in PBS. Brains were extracted and postfixed overnight in 4% PFA at 4°C. Fixed brains were cryoprotected with 30% sucrose in PBS for 2 days at 4°C, and then sectioned on a cryostat (Leica Microsystems GmbH) into 40-μm coronal slices. For immunohistochemical staining, the sections were washed in PBS containing 0.3% Triton X-100 (PBST) twice for 10 min each, incubated in a blocking solution (5% normal donkey serum in PBST) for 1 hour, and incubated with primary antibodies in the blocking solution for 24 to 48 hours at 4°C. The following primary antibodies were used: mouse anti-Cre recombinase (1:200; Millipore, MAB3120), rabbit anti–c-Fos (1:1000; Millipore, ABE457), rabbit anti-PKCδ (1:1000; Abcam, ab182126), rabbit anti-mCherry (1:1000; Abcam, ab167453), and goat anti–green fluorescent protein (1:1000; Abcam, ab6673). After washing in PBST three times for 10 min each, the sections were incubated with secondary antibodies in the blocking solution for 2 hours at room temperature. The following secondary antibodies were used: Alexa 647–labeled donkey anti-rabbit immunoglobulin G (IgG; 1:500; Life Technologies, Carlsbad, CA; A31573), Alexa 594–labeled donkey anti-rabbit IgG (1:500; Life Technologies, A21207), Alexa 488–labeled donkey anti-rabbit IgG (1:500; Life Technologies, A21206), Alexa 488–labeled donkey anti-mouse IgG (1:500; Life Technologies, A21202), and Alexa 488–labeled donkey anti-goat IgG (1:500; Life Technologies, A11055). After washing in PBS three times for 10 min each, the sections were mounted on glass slides using Vectashield hard set mounting medium with 4′,6-diamidino-2-phenylindole (DAPI; H-1500; Vector Laboratories, Burlingame, CA, USA). Fluorescence images were acquired using a confocal laser scanning microscope (FV-10i, Olympus). Cell counting was performed by an experimenter blind to the group allocation using the Cell Counter plugin of the ImageJ software (National Institutes of Health, Bethesda, MD, USA).

To visualize the neurons filled with biocytin during the whole-cell patch-clamp recordings, brain slices were fixed in 4% PFA at 4°C overnight. The slices were washed with PBST twice for 10 min each, incubated in the blocking solution for 1 hour at room temperature, and then incubated with Alexa 647–labeled streptavidin (0.5 μg/ml; Invitrogen, S21374) or Alexa 405–labeled streptavidin (0.5 μg/ml; Invitrogen, S21374) in blocking solution for 48 hours at 4°C. The slices were washed with PBS three times for 10 min each and then mounted on glass slides with Vectashield hard set mounting medium (H-1500; Vector Laboratories). Fluorescence images were acquired using a confocal laser scanning microscope (FV-10i, Olympus).

To examine the overlap between LH-projecting and VTA-projecting adBNST neurons, red and green retrobeads were injected into the LH and VTA, respectively. We also examined the overlap between these neurons by injecting the retrograde-transported AAV2-expressing Cre recombinase into the LH and AAV9-CBA-FLEX-Synaptophysin-mCherry into the adBNST to visualize the nerve terminals of LH-projecting adBNST neurons (*38*). The mice were deeply anesthetized using pentobarbital (100 mg/kg, intraperitoneally) and then transcardially perfused with PBS, followed by 4% PFA in PBS. Brains were extracted and postfixed overnight in 4% PFA at 4°C. Fixed brains were cryoprotected with 30% sucrose in PBS for 2 days at 4°C and then sectioned on a cryostat. The sections were stained with DAPI and mounted on glass slides using Vectashield hard set mounting medium.

### Statistical analyses

Data presented in bar and line graphs indicate means ± SEM. Data presented in box-whisker plots indicate medians, interquartile ranges, and 10th to 90th percentiles. Statistical analyses were conducted using GraphPad Prism (GraphPad Software Inc., La Jolla, CA, USA). Two-tailed unpaired Student’s *t* test, two-tailed paired Student’s *t* test, and two-tailed Mann-Whitney test were used for comparisons between two groups. One-way analysis of variance (ANOVA) with Sidak’s multiple comparison post hoc test was used for comparison among three groups. Two-way ANOVA with Bonferroni’s multiple comparison post hoc test was used for comparison among multiple groups. Chi-square test was used to assess significance for a distribution. Kolmogorov-Smirnov test was used to assess cumulative probability. Differences with *P* < 0.05 were considered significant. Details of the statistical data are provided in table S1.

## Notes

View/request a protocol for this paper from *Bio-protocol*.
