# Supplementary material for: Chronic pain–induced neuronal plasticity in the bed nucleus of the stria terminalis causes maladaptive anxiety
Source: Sci Adv. 2022 Apr 27;8(17):eabj5586. doi: 10.1126/sciadv.abj5586 (PMC9045713; doi:10.1126/sciadv.abj5586)
Supplement: Supplementary file 1 — Figs. S1 to S11 Table S1 [file sciadv.abj5586_sm.pdf]

Science Advances
AAAS

Supplementary Materials for

Chronic pain–induced neuronal plasticity in the bed nucleus of the stria terminalis causes maladaptive anxiety

Naoki Yamauchi, Keiichiro Sato, Kenta Sato, Shunsaku Murakawa, Yumi Hamasaki, Hiroshi Nomura, Taiju Amano, Masabumi Minami*

*Corresponding author. Email: mminami@pharm.hokudai.ac.jp

Published 27 April 2022, *Sci. Adv.* **8**, eabj5586 (2022)
DOI: 10.1126/sciadv.abj5586

The PDF file includes:

Figs. S1 to S11
Table S1
Legend for data S1

Other Supplementary Material for this manuscript includes the following:

Data S1

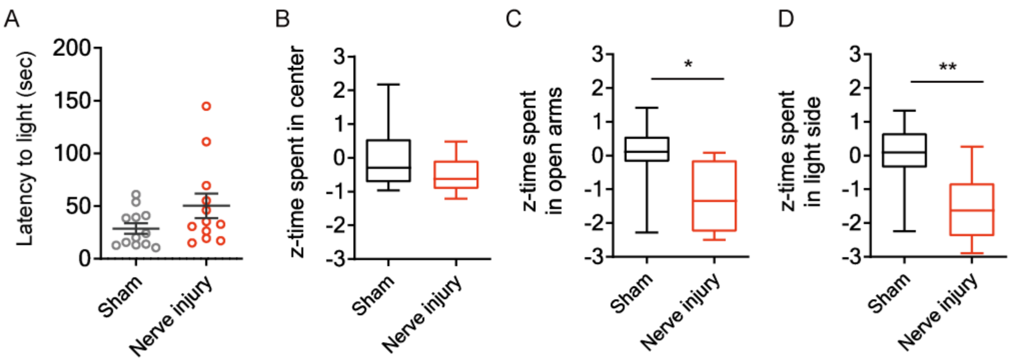

Fig. S1. Chronic pain induces anxiety-like behavior (Emotionality z-scoring). A Scatter plot related to Figure 1F. **B**–**D** z-scoring of the time spent in the center (**B**), time spent in open arms (**C**), and time spent on the light side (**D**). Box-whisker plots show the values of median, interquartile range, and 10th-90th percentiles. Statistical significance was evaluated using two-tailed unpaired Student's *t*-test. $*P < 0.05$, $**P < 0.01$. Details statistical data are provided in Supplementary Table S1.

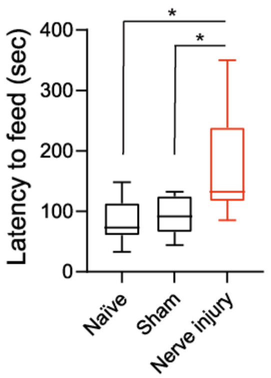

Fig. S2. Chronic pain induces anxiety-like behavior in the NSF test. Anxiety-like behavior was tested by the NSF test. Latency to bite a food pellet was measured in the naïve, sham-operated, and nerve injury groups. Box-whisker plots show the values of median, interquartile range, and 10th-90th percentiles. Statistical significance was evaluated using One-way ANOVA with Sidak's multiple comparison post hoc test. *$P < 0.05$. Details statistical data are provided in Supplementary Table S1.

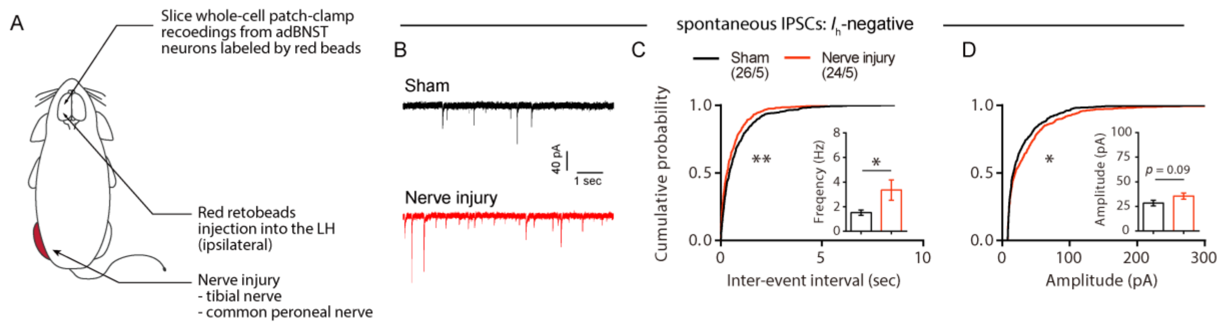

Fig. S3. Chronic pain increases inhibitory synaptic inputs to I_h-negative LH-projecting adBNST neurons ipsilateral to the injury side. A Scheme of experiments. **B** Representative traces of sIPSCs from the ipsilateral I_h-negative LH-projecting adBNST neurons of sham-operated (upper) and nerve injury (lower) mice. **C, D** Cumulative probability plots of the inter-event intervals and the means ± SEM of the sIPSC frequency (**C**) and amplitude (**D**). Kolmogorov-Smirnov test was used to analyze cumulative probability plots. Two-tailed unpaired Student's *t*-test was used to analyze the frequency/amplitude of sIPSCs. $^*P < 0.05$, $^{**}P < 0.01$. Details of statistical data are provided in Supplementary Table S1.

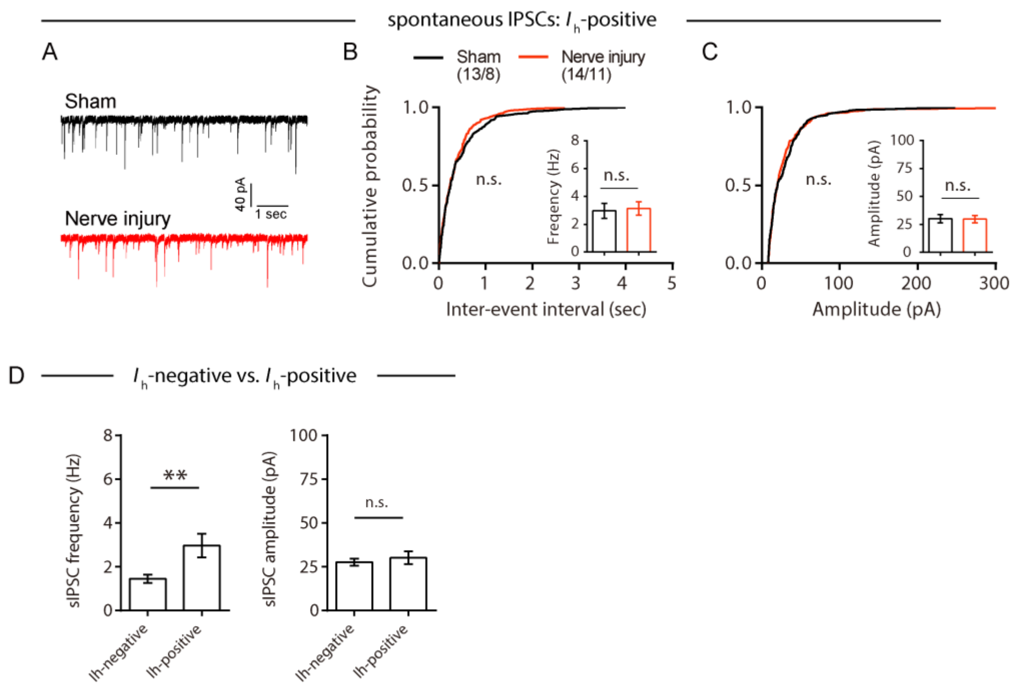

Fig. S4. Chronic pain does not affect inhibitory synaptic inputs to I_h-positive LH-projecting adBNST neurons. A Representative traces of sIPSCs from I_h-positive LH-projecting adBNST neurons of sham-operated (upper) and nerve injury (lower) mice. **B, C** Cumulative probability plots of the inter-event intervals and the means ± SEM of the sIPSC frequency (**B**) and amplitude (**C**). **D** Comparison of sIPSC frequency and amplitude recorded from I_h-positive (n = 13) and I_h-negative (n = 45) neurons of sham-operated mice. Kolmogorov-Smirnov test was used to analyze cumulative probability plots. Two-tailed unpaired Student's t-test was used to analyze the frequency/amplitude of sIPSCs. **$P < 0.01$, n.s.: not significant. The data recorded from contralateral and ipsilateral adBNST neurons were combined. Details of statistical data are provided in Supplementary Table S1.

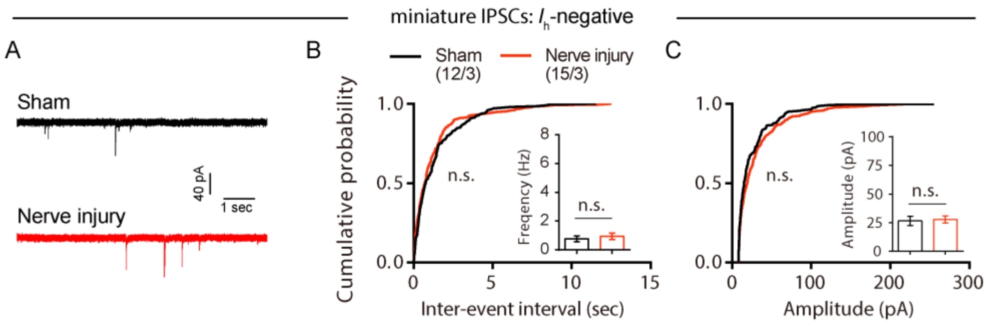

Fig. S5. Increased inhibitory inputs induced by chronic pain disappear in the presence of TTX. A Representative traces of mIPSCs from I_h-negative LH-projecting adBNST neurons of sham-operated (upper) and nerve injury (lower) mice. **B, C** Cumulative probability plots of the inter-event intervals and the means ± SEM of the mIPSC frequency (**B**) and amplitude (**C**). Data were acquired from the contralateral adBNST. Kolmogorov-Smirnov test was used to analyze cumulative probability plots. Two-tailed unpaired Student's *t*-test was used to analyze the frequency/amplitude of mIPSCs. n.s.: not significant. Details of statistical data are provided in Supplementary Table S1.

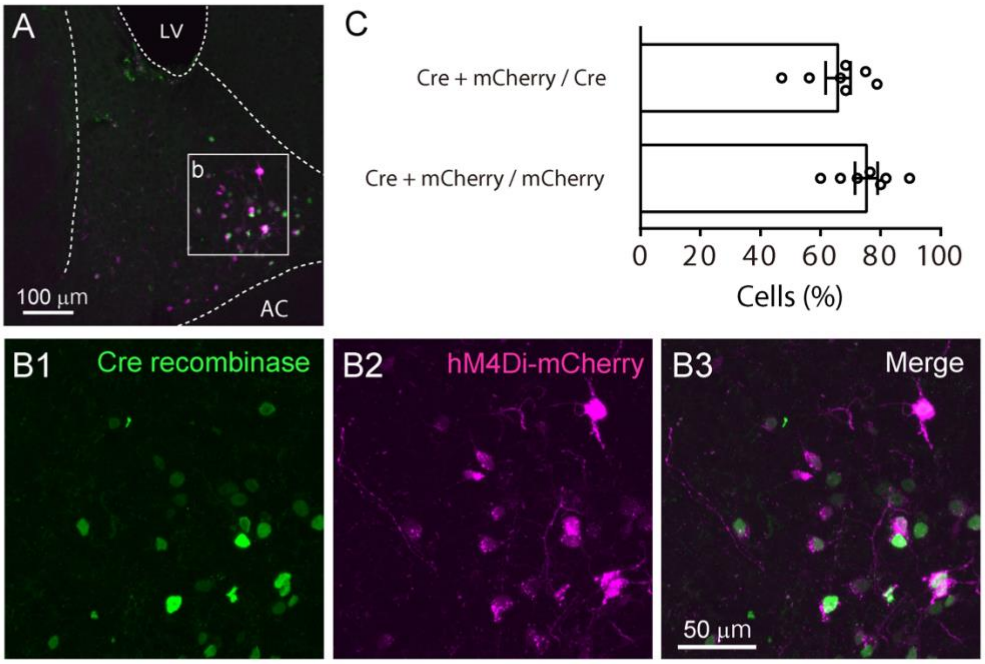

Fig. S6. Cre-dependent expression of hM4Di-mCherry. A, B1-3 Immunostaining of Cre recombinase (green) and hM4Di-mCherry (red) in the adBNST. AAV2 injected into the LH was retrogradely transported, leading to Cre recombinase expression in the adBNST. AAV5 injected into the adBNST Cre-dependently expressed hM4Di-mCherry. Scale bars, 100 µm (**A**) and 50 µm (**B**). **C** Quantitative analyses of specific hM4Di-mCherry expression in the Cre-positive cells (hM4Di-mCherry$^+$/Cre$^+$:75.3 ± 3.7%; Cre$^+$/hM4Di-mCherry$^+$: 65.8 ± 4.1%. *n* = 7 sections from 3 mice. Data are expressed as means ± SEM.

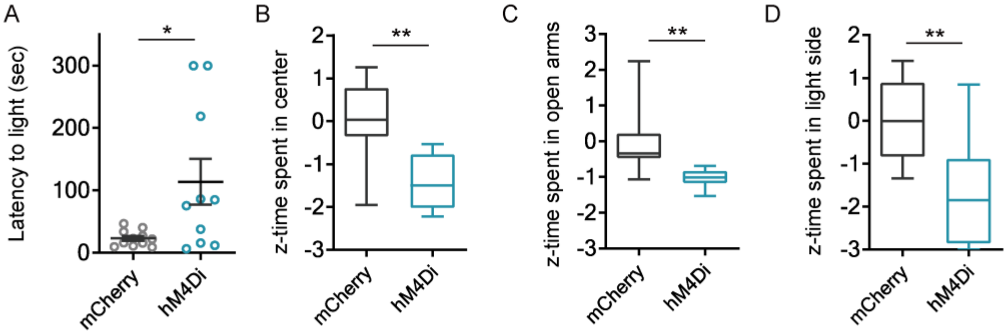

Fig. S7. Inhibition of LH-projecting adBNST neurons induces anxiety-like behavior (Emotionality z-scoring). A Scatter plot related to Figure 3F, showing that the large variability of the latency to light side in the hM4Di group in the LDB test may not be due to the dichotomy within the group. It may be due to the small number of transitions in this group, so that the time to first appearance on the light side varied greatly among the individual animals. **B–D** z-scoring of the time spent in the center (**B**), time spent in open arms (**C**), and time spent on the light side (**D**). Box-whisker plots show the values of median, interquartile range, and 10th-90th percentiles. Statistical significance was evaluated using two-tailed unpaired Student's *t*-test. $^{*}P < 0.05$, $^{**}P < 0.01$. Details of statistical data are provided in Supplementary Table S1.

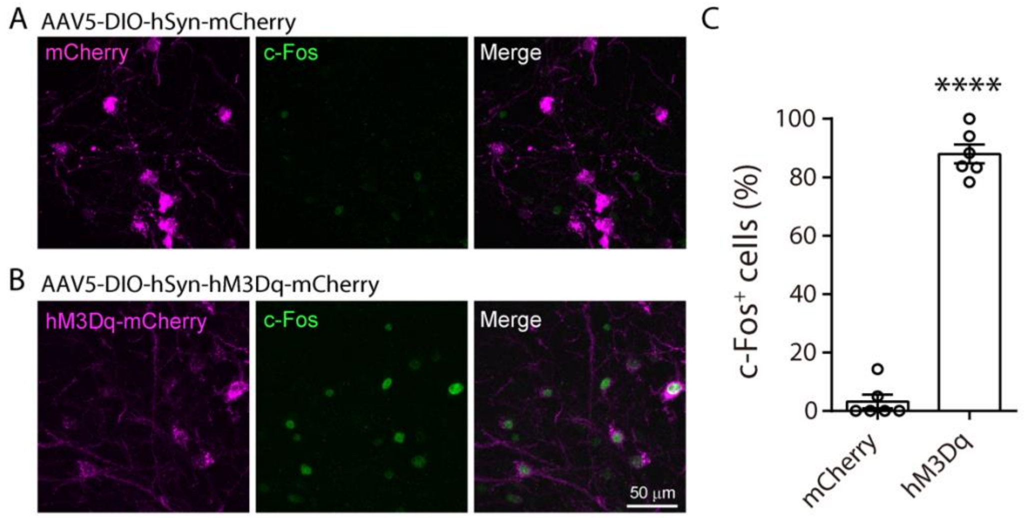

Fig. S8. Functional expression of hM3Dq-mCherry in the adBNST. A, B Representative confocal images showing c-Fos expression (green) in mCherry-expressing (**A**) or hM3Dq-mCherry-expressing (**B**) cells (red) in the adBNST of nerve injury mice. Mice were sacrificed and their brains were removed 180 min after CNO administration (1 mg/kg, intraperitoneally). c-Fos and mCherry were visualized by immunostaining. Scale bar; 50 µm. C Quantitative analyses of c-Fos expression in the adBNST of nerve injury mice. Greater c-Fos expression was observed in the cells expressing hM3Dq-mCherry (n = 6 sections from 2 mice) compared to those expressing mCherry alone (n = 6 sections from 2 mice). Data are expressed as means ± SEM. Statistical significance was evaluated using two-tailed unpaired Student's t-test. ****$P < 0.0001$. Details of statistical data are provided in Supplementary Table S1.

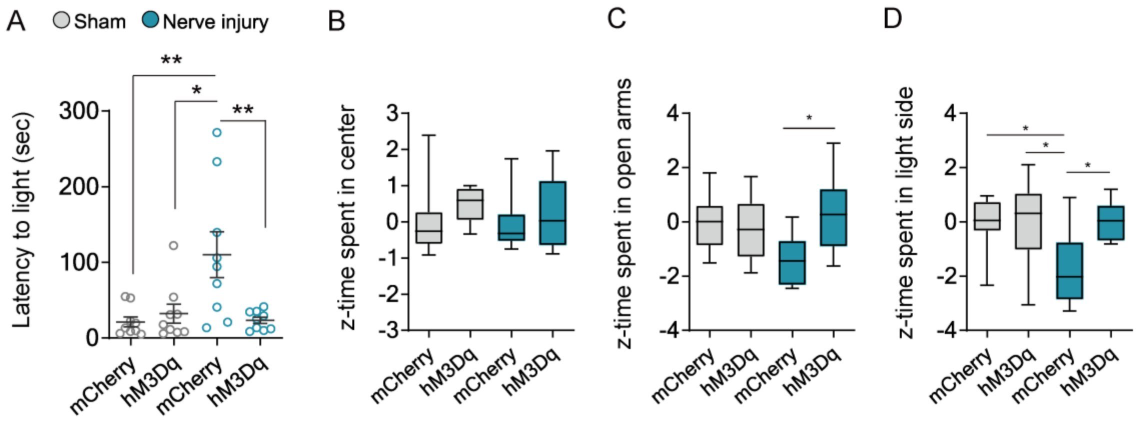

Fig. S9. Activation of LH-projecting adBNST neurons ameliorates chronic pain-induced anxiety-like behavior (Emotionality z-scoring). A Scatter plot related to Figure 4F, showing that the large variability of the latency to light side in the mCherry-expressing nerve injury group in the LDB test may not be due to the dichotomy within the group. It may be due to the small number of transitions in this group, so that the time to first appearance on the light side varied greatly among individual animals. **B–D** z-scoring of the time spent in the center (**B**), time spent in open arms (**C**), and time spent in the light side (**D**). Box-whisker plots show the values of median, interquartile range, and 10th-90th percentiles. Statistical significance was evaluated using two-tailed unpaired Student's *t*-test. $*P < 0.05$, $**P < 0.01$. Details of statistical data are provided in Supplementary Table S1.

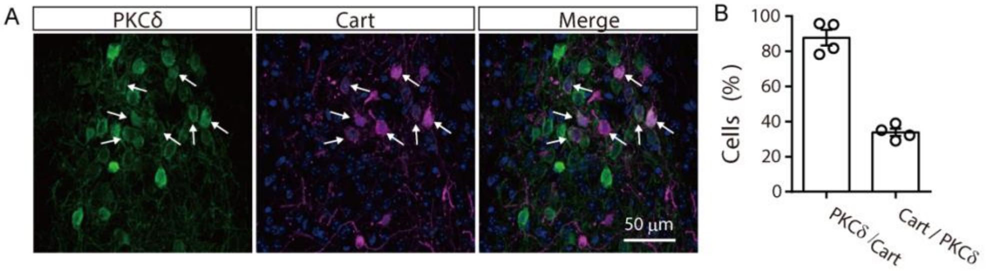

Fig. S10. CART-positive BNST neurons are a subset of PKCδ-positive BNST neurons. A Immunostaining of PKCδ (green) and viral expression of mCherry (magenta) in the ovBNST. Arrows indicate neurons expressing both PKCδ and mCherry. Scale bar, 50 µm. **B** Quantification of overlap (*n* = 4 mice). Data are expressed as means ± SEM.

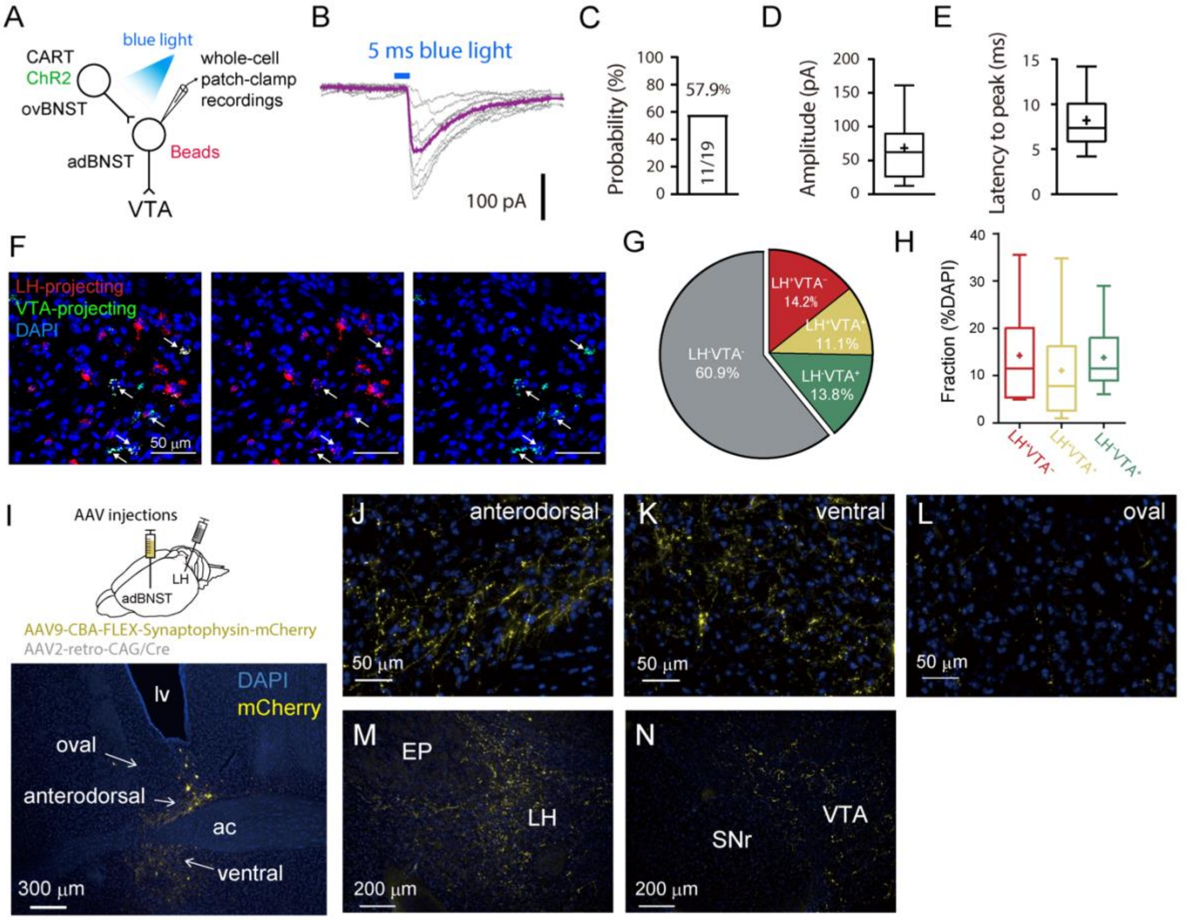

Fig. S11. VTA-projecting adBNST neurons also receive inhibitory inputs from CART-expressing BNST neurons and partially overlap LH-projecting adBNST neurons. A Scheme of experiments. AAV9 delivering Cre-dependent ChR2 expression construct was injected into the ovBNST of Cart-Cre mouse, and then red retrobeads were injected into the VTA. Whole-cell patch-clamp recordings from retrobeads-labeled adBNST neurons were performed to examine the electrophysiological responses to blue-light stimulation. **B** Representative trace showing light-evoked IPSCs. Functional synaptic connectivity was defined to be present when evoked IPSCs with an amplitude of 10 pA or greater were observed in at least 60% of the light stimulation trials (6/10 stimulations). **C** Synaptic connectivity from ovBNST^{CART} neurons to I_h-negative VTA-projecting adBNST neurons. Eleven out of 19 cells from 2 mice showed light-evoked IPSCs. **D, E** The amplitude (**D**) and latency to peak (**E**) in the recorded neurons (n = 11 cells from 2 mice). Box-whisker plots indicate median, interquartile range, and 10th-90th percentiles. Means are indicated by "+" in (**D**) and (**E**). **F** Representative confocal images showing the distribution of LH-projecting (red) and VTA-projecting (green) neurons in the BNST. White arrows indicate the neurons projecting to both the LH and VTA. Scale bars, 50 µm. **G, H** Proportion of LH-projecting neurons, VTA-projecting neurons, and the neurons projecting to both the LH and VTA among total adBNST neurons (DAPI-positive neurons). Box-whisker plots indicate median, interquartile range, and 10th-90th percentiles. Means are indicated by "+" in (**H**). n = 8 mice. **I, upper** AAV2-retro/CAG-Cre and AAV9-CBA-FLEX-Synaptophysin-mCherry were injected into the LH and adBNST, respectively, to visualize the

nerve terminals of LH-projecting adBNST neurons. **I**‑**N** Representative confocal images showing the distribution of Synaptophysin-mCherry in the BNST (**I, lower**), anterodorsal BNST (**J**), ventral BNST (**K**), oval BNST (**L**), LH (**M**) and VTA (**N**). Scale bars, 300 µm (**I, lower**), 50 µm (**J–L**), and 200 µm (**M, N**). ac: anterior commissure, lv: lateral ventricle, EP: entopeduncular nucleus, SNr: substantia nigra, reticular part.

Table S1. Details of statistical data

Fig No.		Experimental group	N	Test	Comparison	F(DFn,DFd) = F-value	P-value	
	**von Frey**							
Figure 1C	Mechanical threshold	Sham-ipsi; Sham-contra; Nerve injury-ipsi; Nerve injury-contra	23; 23; 22; 22	RM TW-ANOVA	Interaction	F (12, 344) = 21.12	< 0.0001	****
					Time point	F (4, 344) = 32.02	< 0.0001	****
					Model	F (3, 86) = 182.6	< 0.0001	****

post-hoc group	P-value									
	**pre**		**1 w**		**2 w**		**3w**		**4 w**	
Nerve injury-ipsilateral vs. Nerve injury-contralateral	0.8896	ns	< 0.0001	****	< 0.0001	****	< 0.0001	****	< 0.0001	****
Nerve injury-ipsilateral vs. Sham-ipsilateral	0.9838	ns	< 0.0001	****	< 0.0001	****	< 0.0001	****	< 0.0001	****
Nerve injury-ipsilateral vs. Sham-contralaterall	0.6687	ns	< 0.0001	****	< 0.0001	****	< 0.0001	****	< 0.0001	****
Nerve injury-contralateral vs. Sham-ipsilateral	0.6967	ns	0.5982	ns	0.5568	ns	0.0263	*	0.4898	ns
Nerve injury-contralateral vs. Sham-contralaterall	0.2467	ns	0.8544	ns	0.0201	*	0.0042	**	0.3793	ns
Sham-ipsilateral vs. Sham-contralaterall	0.8632	ns	0.9695	ns	0.3729	ns	0.9361	ns	0.9977	ns

Fig No.		Experimental group	N	Test	Comparsion	F(DFn,DFd) = F-value	P-value	
	**OFT**							
Figure 1D	Time in center	Sham; Nerve injury	20;19	Unpaired t test	Sham vs. Nerve injury	t=2.022, df=37	0.0504	ns
	Center entry	Sham; Nerve injury	20;19	Unpaired t test	Sham vs. Nerve injury	t=2.104, df=37	0.0422	*
	Distance	Sham; Nerve injury	20;19	Unpaired t test	Sham vs. Nerve injury	t=1.366, df=37	0.1801	ns
	**EPM**							
Figure 1E	Time in open arm	Sham; Nerve injury	19;18	Unpaired t test	Sham vs. Nerve injury	t=3.285 df=27	0.0028	**
	open arm entry	Sham; Nerve injury	19;18	Unpaired t test	Sham vs. Nerve injury	t=2.408 df=27	0.0232	*
	Distance	Sham; Nerve injury	19;18	Unpaired t test	Sham vs. Nerve injury	t=1.010 df=27	0.3213	ns
	Distance in open arms / total distance	Sham; Nerve injury	19;18	Unpaired t test	Sham vs. Nerve injury	t=2.490, df=35	0.0177	*
	**LDB**							
Figure 1F	Time in light side	Sham; Nerve injury	12;12	Unpaired t test	Sham vs. Nerve injury	t=2.156 df=22	0.0423	*
	Latency to light	Sham; Nerve injury	12;12	Unpaired t test	Sham vs. Nerve injury	t=1.705 df=22	0.1022	ns
	Transitions	Sham; Nerve injury	12;12	Unpaired t test	Sham vs. Nerve injury	t=1.728 df=22	0.098	ns
	**z-score**							
Figure 1G	Emotionality score	Sham; Nerve injury	9;9	Unpaired t test	Sham vs. Nerve injury	t=4.090, df=16	0.0009	***

Fig No.		Experimental group	N	Test	Comparison	F(DFn,DFd) = F-value	P-value	
Figure 2C	Proportion	Sham; Nerve injury	104; 94	Chi-square test	sham vs. Nerve injury	χ^2 (1)= 0.2996	0.5841	ns
Figure 2E	sIPSC frequency	Sham; Nerve injury	19; 20	Kolmogorov-Smirnov test	sham vs. Nerve injury	D=0.3034199	< 0.0001	****
				Unpaired t test	sham vs. Nerve injury	t=3.174 df=37	0.003	**
Figure 2F	sIPSC amplitude	Sham; Nerve injury	19;20	Kolmogorov-Smirnov test	sham vs. Nerve injury	D=0.1467151	0.0006	***
				Unpaired t test	sham vs. Nerve injury	t=2.142 df=37	0.0389	*
Figure 2H	sEPSC frequency	Sham; Nerve injury	26;13	Kolmogorov-Smirnov test	sham vs. Nerve injury	D=0.2016484	< 0.0001	****
				Mann-Whitney U test	sham vs. Nerve injury	U=107	0.0654	ns
Figure 2I	sEPSC amplitude	Sham; Nerve injury	26;13	Kolmogorov-Smirnov test	sham vs. Nerve injury	D=0.09175824	0.0933	ns
				Unpaired t test	sham vs. Nerve injury	t=0.5070 df=37	0.6152	ns

Fig No.		Experimental group	*N*	Test	Comparison	F(DFn,DFd) = F-value	P-value	
Figure 3C	Resting membrane potential	hM4Di	4	Paired t-test	baseline vs. CNO	t=3.386 df=5	0.0195	*
	Membrane resistance	hM4Di	4	Paired t-test	baseline vs. CNO	t=3.494 df=5	0.0174	*
	**OFT**							
Figure 3D	Time in center	mCherry; hM4Di	11;10	Unpared t-test	mCherry vs. hM4Di	t=3.166 df=19	0.0051	**
	Center entry	mCherry; hM4Di	11;10	Unpared t-test	mCherry vs. hM4Di	t=3.347 df=19	0.0034	**
	Distance	mCherry; hM4Di	11;10	Unpared t-test	mCherry vs. hM4Di	t=1.935 df=19	0.068	ns
	**EPM**							
Figure 3E	Time in open arms	mCherry; hM4Di	11;10	Unpared t-test	mCherry vs. hM4Di	t=3.903 df=19	0.001	**
	Open arm entry	mCherry; hM4Di	11;10	Unpared t-test	mCherry vs. hM4Di	t=1.144 df=19	0.267	
	Distance	mCherry; hM4Di	11;10	Unpared t-test	mCherry vs. hM4Di	t=2.362 df=19	0.029	*
	Distance in open arms / total distance	mCherry; hM4Di	11;10	Unpared t-test	mCherry vs. hM4Di	t=2.783 df=19	0.0118	*
	**LDB**							
Figure 3F	Time in light	mCherry; hM4Di	11;10	Unpared t-test	mCherry vs. hM4Di	t=3.410 df=19	0.0029	**
	Latency to light	mCherry; hM4Di	11;10	Unpared t-test	mCherry vs. hM4Di	t=2.586 df=19	0.0181	*
	Transition	mCherry; hM4Di	11;10	Unpared t-test	mCherry vs. hM4Di	t=5.025 df=19	< 0.0001	****
	**z-score**							
Figure 3G	Emotionality score	mCherry; hM4Di	11;10	Unpared t-test	mCherry vs. hM4Di	t=6.323, df=19	< 0.0001	****
	**von Frey test**							
Figure 3H	Mechanical threshold	mCherry; hM4Di	22; 20	Mann-Whitney U test	mCherry vs. hM4Di	U = 200.5	0.6268	ns

Fig No.		Experimental group	*N*	Test	Comparison	F(DFn,DFd) = F-value	P-value	
	**OFT**							
Figure 4D	Time in center	sham-mCherry; sham-hM3Dq; Nerve injury-mCherry; Nerve injury-hM3Dq	9;9;9;9	TW-ANOVA	Interaction	F (1, 32) = 0.1696	0.6832	ns
					Dreadd	F (1, 32) = 1.842	0.1842	ns
					Model	F (1, 32) = 0.2984	0.5887	ns
	Center entry	sham-mCherry; sham-hM3Dq; Nerve injury-mCherry; Nerve injury-hM3Dq	9;9;9;9	TW-ANOVA	Interaction	F (1, 32) = 0.2150	0.646	ns
					Dreadd	F (1, 32) = 5.893	0.021	*
					Model	F (1, 32) = 3.339	0.077	ns
	Distance	sham-mCherry; sham-hM3Dq; Nerve injury-mCherry; Nerve injury-hM3Dq	9;9;9;9	TW-ANOVA	Interaction	F (1, 32) = 0.01713	0.8967	ns
					Dreadd	F (1, 32) = 6.776	0.0139	*
					Model	F (1, 32) = 2.192	0.1485	ns
	**EPM**							
Figure 4E	Time in open arm	sham-mCherry; sham-hM3Dq; Nerve injury-mCherry; Nerve injury-hM3Dq	9;9;9;9	TW-ANOVA	Interaction	F (1, 32) = 6.590	0.0249	*
					Dreadd	F (1, 32) = 3.337	0.0893	ns
					Model	F (1, 32) = 1.305	0.3132	ns

post-hoc group	P-value	
mCherry:Sham vs. mCherry:Nerve injury	0.0794	ns
mCherry:Sham vs. hM3Dq:Sham	>0.9999	ns
mCherry:Sham vs. hM3Dq:Nerve injury	>0.9999	ns
mCherry:Nerve injury vs. hM3Dq:Sham	0.2624	ns
mCherry:Nerve injury vs. hM3Dq:Nerve injury	0.0237	*
hM3Dq:Sham vs. hM3Dq:Nerve injury	>0.9999	ns

Fig No.		Experimental group	*N*	Test	Comparision	F(DFn,DFd) = F-value	P-value	
Figure 4E	open arm entry	sham-mCherry; sham-hM3Dq; Nerve injury-mCherry; Nerve injury-hM3Dq	9;9;9;9	TW-ANOVA	Interaction	F (1, 32) = 3.130	0.0864	ns
					Dreadd	F (1, 32) = 2.491	0.1243	ns
					Model	F (1, 32) = 4.225	0.0481	*
	Distance	sham-mCherry; sham-hM3Dq; Nerve injury-mCherry; Nerve injury-hM3Dq	9;9;9;9	TW-ANOVA	Interaction	F (1, 32) = 0.7922	0.3801	ns
					Dreadd	F (1, 32) = 15.15	0.0005	***
					Model	F (1, 32) = 0.3549	0.5555	ns
	**LDB**							
Figure 4F	Time in light side	sham-mCherry; sham-hM3Dq; Nerve injury-mCherry; Nerve injury-hM3Dq	9;9;9;9	TW-ANOVA	Interaction	F (1, 32) = 5.140	0.0303	*
					Dreadd	F (1, 32) = 4.053	0.0526	ns
					Model	F (1, 32) = 4.219	0.0482	*

post-hoc group	P-value	
mCherry:Sham vs. mCherry:Nerve injury	0.027	*
mCherry:Sham vs. hM3Dq:Sham	>0.9999	ns
mCherry:Sham vs. hM3Dq:Nerve injury	>0.9999	ns
mCherry:Nerve injury vs. hM3Dq:Sham	0.0426	*
mCherry:Nerve injury vs. hM3Dq:Nerve injury	0.0291	*
hM3Dq:Sham vs. hM3Dq:Nerve injury	>0.9999	ns

	Latency to light	sham-mCherry; sham-hM3Dq; Nerve injury-mCherry; Nerve injury-hM3Dq	9;9;9;9	TW-ANOVA	Interaction	F (1, 32) = 8.475	0.0065	**
					Dreadd	F (1, 32) = 5.139	0.0303	*
					Model	F (1, 32) = 5.687	0.0232	*

post-hoc group	P-value	
mCherry:Sham vs. mCherry:Nerve injury	0.0043	**
mCherry:Sham vs. hM3Dq:Sham	>0.9999	ns
mCherry:Sham vs. hM3Dq:Nerve injury	>0.9999	ns
mCherry:Nerve injury vs. hM3Dq:Sham	0.0147	*
mCherry:Nerve injury vs. hM3Dq:Nerve injury	0.0054	**
hM3Dq:Sham vs. hM3Dq:Nerve injury	>0.9999	ns

	Transitions	sham-mCherry; sham-hM3Dq; Nerve injury-mCherry; Nerve injury-hM3Dq	9;9;9;9	TW-ANOVA	Interaction	F (1, 32) = 6.582	0.0152	*
					Dreadd	F (1, 32) = 4.977	0.0328	*
					Model	F (1, 32) = 2.799	0.1041	ns

post-hoc group	P-value	
mCherry:Sham vs. mCherry:Nerve injury	0.0314	*
mCherry:Sham vs. hM3Dq:Sham	>0.9999	ns
mCherry:Sham vs. hM3Dq:Nerve injury	>0.9999	ns
mCherry:Nerve injury vs. hM3Dq:Sham	0.0568	ns
mCherry:Nerve injury vs. hM3Dq:Nerve injury	0.0112	*
hM3Dq:Sham vs. hM3Dq:Nerve injury	>0.9999	ns

	**z-score**							
Figure 4G	Emotionality score	sham-mCherry; sham-hM3Dq; Nerve injury-mCherry; Nerve injury-hM3Dq	9;9;9;9	TW-ANOVA	Interaction	F (1, 32) = 8.118	0.0076	**
					Dreadd	F (1, 32) = 8.877	0.0055	**
					Model	F (1, 32) = 4.971	0.0329	*

post-hoc group	P-value	
mCherry:Sham vs. mCherry:Nerve injury	0.0065	**
mCherry:Sham vs. hM3Dq:Sham	>0.9999	ns
mCherry:Sham vs. hM3Dq:Nerve injury	>0.9999	ns
mCherry:Nerve injury vs. hM3Dq:Sham	0.0051	**
mCherry:Nerve injury vs. hM3Dq:Nerve injury	0.0015	**
hM3Dq:Sham vs. hM3Dq:Nerve injury	>0.9999	ns

Fig No.		Experimental group	*N*	Test	Comparision	F(DFn,DFd) = F-value	P-value
	**von Frey**						
Figure 4H	Mechanical threshold	mCherry-Pre; mCherry-CNO; hM3Dq-Pre; hM3Dq-CNO	9;9	RM TW-ANOVA	Interaction	F (1, 16) = 4.004	0.0626
					Dreadd	F (1, 16) = 6.226	0.0239
					CNO	F (1, 16) = 2.171	0.1601

Fig No.		Experimental group	*N*	Test	Comparison	F(DFn,DFd) = F-value	P-value	
Figure 6B	Resting membrane potential	Sham; Nerve injury	20;22	Unpaired t test	Sham vs. Nerve injury	t=1.119764, df=40	0.2695	ns
Figure 6C	Rheobase	Sham; Nerve injury	20;22	Unpaired t test	Sham vs. Nerve injury	t=3.490638, df=40	0.0012	**
	Action potential threshold	Sham; Nerve injury	20;22	Unpaired t test	Sham vs. Nerve injury	t=2.735135, df=40	0.0092	**
Figure 6D	Membrane resistance	Sham; Nerve injury	20;22	Unpaired t test	Sham vs. Nerve injury	t=2.147873, df=40	0.0378	*
Figure 6E	Action potential amplitude	Sham; Nerve injury	20;22	Unpaired t test	Sham vs. Nerve injury	t=2.573846, df=40	0.0139	*

Fig No.		Experimental group	*N*	Test	Comparison	F(DFn,DFd) = F-value	P-value	
Figure 7C	sIPSC frequency	sham-hM4Di; Nerve injury-EYFP; Nerve injury-hM4Di	10;10;13	RM TW-ANOVA	Interaction	F (2, 30) = 9.365	0.0007	***
					Model	F (2, 30) = 2.723	0.0819	ns
					CNO	F (1, 30) = 5.817	0.0222	*

post-hoc group	P-value	
sham-hM4Di	0.9695	ns
Nerve injury-EYFP	0.7528	ns
Nerve injury-hM4Di	<0.0001	****

Fig No.		Experimental group	*N*	Test	Comparison	F(DFn,DFd) = F-value	P-value	
Figure 7D	ΔsIPSC frequency	sham-hM4Di; Nerve injury-EYFP; Nerve injury-hM4Di	10;10;13	OW-ANOVA	Interaction	F (2, 30) = 9.365	0.0007	***

post-hoc group	P-value	
sham-hM4Di vs. Nerve injury-EYFP	0.7391	ns
sham-hM4Di vs. Nerve injury-hM4Di	0.0129	*
Nerve injury-EYFP vs. Nerve injury-hM4Di	0.0009	***

Fig No.		Experimental group	*N*	Test	Comparison	F(DFn,DFd) = F-value	P-value	
	**EPM**							
Figure 7E	Time in open arm	mCherry; Nerve injury	8;7	Unpaired t test	Sham vs. Nerve injury	t=2.399741, df=13	0.0321	*
	Open arm entry	mCherry; Nerve injury	8;7	Unpaired t test	Sham vs. Nerve injury	t=1.296735, df=13	0.2173	ns
	Distance	mCherry; Nerve injury	8;7	Unpaired t test	Sham vs. Nerve injury	t=2.573613, df=13	0.0231	*

Fig No.		Experimental group	*N*	Test	Comparison	F(DFn,DFd) = F-value	P-value	
Figure S1B	z-time spent in center	Sham; Nerve injury	9;9	Unpaired t test	Sham vs. Nerve injury	t=1.356, df=16	0.194	n.s.
Figure S1C	z-time spent in open arms	Sham; Nerve injury	9;9	Unpaired t test	Sham vs. Nerve injury	t=2.730, df=16	0.0148	*
Figure S1D	z-time spent in light side	Sham; Nerve injury	9;9	Unpaired t test	Sham vs. Nerve injury	t=3.334, df=16	0.0042	**

Fig No.		Experimental group	*N*	Test	Comparison	F(DFn,DFd) = F-value	P-value	
Figure S2	Latency to feed	Naïve; Sham; Nerve injury	8;8;8	OW-ANOVA	Interaction	F (2, 21) = 6.006	0.0087	**

post-hoc group	P-value	
Naïve vs. Sham	>0.9999	ns
Naïve vs. Nereve injury	0.0142	*
Sham vs. Nerve injury	0.0305	*

Fig No.		Experimental group	*N*	Test	Comparison	F(DFn,DFd) = F-value	P-value	
Figure S3C	sIPSC frequency	Sham; Nerve injury	26;24	Kolmogorov-Smirnov test	sham vs. Nerve injury	D=0.09183743	0.0036	**
				Unpaired t test	sham vs. Nerve injury	t=2.242 df=48	0.0296	*
Figure S3D	sIPSC amplitude	Sham; Nerve injury	26;24	Kolmogorov-Smirnov test	sham vs. Nerve injury	D=0.1113614	0.0158	*
				Unpaired t test	sham vs. Nerve injury	t=1.716 df=48	0.0927	ns

Fig No.		Experimental group	*N*	Test	Comparison	F(DFn,DFd) = F-value	P-value	
Figure S4B	sIPSC frequency	Sham; Nerve injury	13;14	Kolmogorov-Smirnov test	sham vs. Nerve injury	D=0.06887755	0.5649	ns
				Unpaired t test	sham vs. Nerve injury	t=0.2268 df=25	0.8224	ns
Figure S4C	sIPSC amplitude	Sham; Nerve injury	13;14	Kolmogorov-Smirnov test	sham vs. Nerve injury	D=0.08410138	0.31	ns
				Unpaired t test	sham vs. Nerve injury	t=0.09728 df=25	0.9233	ns
Figure S4D	sIPSC frequency	Ih-negative; Ih-positive	45; 13	Unpaired t test	Ih-negative vs. Ih-positive	t=3.347, df=56	0.0015	**
	sIPSC amplitude	Ih-negative; Ih-positive	45; 13	Unpaired t test	Ih-negative vs. Ih-positive	t=0.6046, df=56	0.5479	ns

Fig No.		Experimental group	*N*	Test	Comparison	F(DFn,DFd) = F-value	P-value	
Figure S5B	mIPSC frequency	Sham; Nerve injury	12;15	Kolmogorov-Smirnov test	sham vs. Nerve injury	D=0.08874167	0.3496	ns
				Unpaired t test	sham vs. Nerve injury	t=0.5477 df=25	0.5888	ns
Figure S5C	mIPSC amplitude	Sham; Nerve injury	12;15	Kolmogorov-Smirnov test	sham vs. Nerve injury	D=0.1150779	0.08	ns
				Unpaired t test	sham vs. Nerve injury	t=0.2720 df=25	0.7879	ns

Fig No.		Experimental group	*N*	Test	Comparsion	F(DFn,DFd) = F-value	P-value	
Figure S7B	z-time spent in center	mCherry; hM4Di	11;10	Unpared t-test	mCherry vs. hM4Di	t=3.166 df=19	0.0051	**
Figure S7C	z-time spent in open arms	mCherry; hM4Di	11;10	Unpared t-test	mCherry vs. hM4Di	t=3.903 df=19	0.001	**
Figure S7D	z-time spent in light side	mCherry; hM4Di	11;10	Unpared t-test	mCherry vs. hM4Di	t=3.410 df=19	0.0029	**

Fig No.		Experimental group	*N*	Test	Comparison	F(DFn,DFd) = F-value	P-value	
Figure S8C	c-Fos+ cells	mCherry; hM3Dq	6; 6	Unpaired t test	mCherry vs. hM3Dq	t=21.16, df=10	<0.0001	****

Fig No.		Experimental group	*N*	Test	Comparison	F(DFn,DFd) = F-value	P-value	
	**OFT**							
Figure S9B	z-time in center	sham-mCherry; sham-hM3Dq; Nerve injury-mCherry; Nerve injury-hM3Dq	9;9;9;9	TW-ANOVA	Interaction	F (1, 32) = 0.1696	0.6832	ns
					Dreadd	F (1, 32) = 1.842	0.1842	ns
					Model	F (1, 32) = 0.2984	0.5887	ns
	**EPM**							
Figure S9C	z-time in open arm	sham-mCherry; sham-hM3Dq; Nerve injury-mCherry; Nerve injury-hM3Dq	9;9;9;9	TW-ANOVA	Interaction	F (1, 32) = 6.590	0.0249	*
					Dreadd	F (1, 32) = 3.337	0.0893	ns
					Model	F (1, 32) = 1.305	0.3132	ns

post-hoc group	P-value	
mCherry:Sham vs. mCherry:Nerve injury	0.0794	ns
mCherry:Sham vs. hM3Dq:Sham	>0.9999	ns
mCherry:Sham vs. hM3Dq:Nerve injury	>0.9999	ns
mCherry:Nerve injury vs. hM3Dq:Sham	0.2624	ns
mCherry:Nerve injury vs. hM3Dq:Nerve injury	0.0237	*
hM3Dq:Sham vs. hM3Dq:Nerve injury	>0.9999	ns

Fig No.		Experimental group	*N*	Test	Comparision	F(DFn,DFd) = F-value	P-value	
	**LDB**							
Figure S9D	z-time in light side	sham-mCherry; sham-hM3Dq; Nerve injury-mCherry; Nerve injury-hM3Dq	9;9;9;9	TW-ANOVA	Interaction	F (1, 32) = 5.140	0.0303	*
					Dreadd	F (1, 32) = 4.053	0.0526	ns
					Model	F (1, 32) = 4.219	0.0482	*

post-hoc group	P-value	
mCherry:Sham vs. mCherry:Nerve injury	0.027	*
mCherry:Sham vs. hM3Dq:Sham	>0.9999	ns
mCherry:Sham vs. hM3Dq:Nerve injury	>0.9999	ns
mCherry:Nerve injury vs. hM3Dq:Sham	0.0426	*
mCherry:Nerve injury vs. hM3Dq:Nerve injury	0.0291	*
hM3Dq:Sham vs. hM3Dq:Nerve injury	>0.9999	ns

Data S1. Individual data related to Figures 1 to 7 and Supplementary Figures S1 to S11
